# Cost effectiveness of mHealth intervention by community health workers for reducing maternal and newborn mortality in rural Uttar Pradesh, India

**DOI:** 10.1186/s12962-018-0110-2

**Published:** 2018-06-25

**Authors:** Shankar Prinja, Pankaj Bahuguna, Aditi Gupta, Ruby Nimesh, Madhu Gupta, Jarnail Singh Thakur

**Affiliations:** School of Public Health, Post Graduate Institute of Medical Education and Research, Sector-12, Chandigarh, 160012 India

**Keywords:** mHealth, Cost effectiveness, Maternal and child health, DALY

## Abstract

**Background:**

A variety of mobile-based health technologies (mHealth) have been developed for use by community health workers to augment their performance. One such mHealth intervention—ReMiND program, was implemented in a poor performing district of India. Despite some research on the extent of its effectiveness, there is significant dearth of evidence on cost-effectiveness of such mHealth interventions. In this paper we evaluated the incremental cost per disability adjusted life year (DALY) averted as a result of ReMiND intervention as compared to routine maternal and child health programs without ReMiND.

**Methods:**

A decision tree was parameterized on MS-Excel spreadsheet to estimate the change in DALYs and cost as a result of implementing ReMiND intervention compared with routine care, from both health system and societal perspective. A time horizon of 10 years starting from base year of 2011 was considered appropriate to cover all costs and effects comprehensively. All costs, including those during start-up and implementation phase, besides other costs on the health system or households were estimated. Consequences were measured as part of an impact assessment study which used a quasi-experimental design. Proximal outputs in terms of changes in service coverage were modelled to estimate maternal and infant illnesses and deaths averted, and DALYs averted in Uttar Pradesh state of India. Probabilistic sensitivity analysis was undertaken to account for parameter uncertainties.

**Results:**

Cumulatively, from year 2011 to 2020, implementation of ReMiND intervention in UP would result in a reduction of 312 maternal and 149,468 neonatal deaths. This implies that ReMiND program led to a reduction of 0.2% maternal and 5.3% neonatal deaths. Overall, ReMiND is a cost saving intervention from societal perspective. From health system perspective, ReMiND incurs an incremental cost of INR 12,993 (USD 205) per DALY averted and INR 371,577 (USD 5865) per death averted.

**Conclusions:**

Overall, findings of our study suggest strongly that the mHealth intervention as part of ReMiND program is cost saving from a societal perspective and should be considered for replication elsewhere in other states.

**Electronic supplementary material:**

The online version of this article (10.1186/s12962-018-0110-2) contains supplementary material, which is available to authorized users.

## Background

The state of Uttar Pradesh (UP) is one of the major contributors to maternal and child deaths in India with low coverage of key maternal, neonatal and child health (MNCH) services [1]. The coverage of institutional deliveries, full antenatal care (ANC) and full immunization in UP were 45.6, 29.6, 45.3% respectively in year 2011–2012 [2]. Kaushambi is one of the 19 high focus districts in UP and exhibits some of the worst health statistics with maternal mortality ratio and infant mortality rate being 366 per 100,000 live births and 80 deaths per 1000 live births respectively [2, 3].

In 2005, National Rural Health Mission (NRHM)—now called National Health Mission (NHM) was introduced in India with the purpose of improving various health indicators through strengthening of government health care system [1]. A new cadre of community health workers called Accredited Social Health Activist (ASHA) was introduced to generate public demand for health services [4]. ASHAs are the local women who act as community mobilizer and motivator with a prime purpose to generate demand for health care services and to serve as a link between health system and community, primarily for maternal and child health services. An evaluation of ASHAs in year 2011 found that although a 23-day training schedule has been developed by Ministry of Health and Family Welfare (MoHFW), but the quality of training needs to be strengthened in order to improve their performance [5].

To bolster and supplement the knowledge of community health workers in developing countries, mobile technology is being utilized as one of the effective and sustainable method [6]. Several positive effects of use of mHealth interventions have been noted in literature. Knowledge about the number of ANC visits among community showed an increase from 10 to 37% after introduction of text messages for health promotion in southern Indian state of Tamil Nadu [7]. Use of mHealth intervention in Afghanistan resulted in 20% improvement in antenatal attendance and 22.3% improvement in the number of women receiving skilled deliveries at a health facility [8]. In terms of quality of counselling, a study from India found increase in knowledge retention of health workers about identification of danger signs during pregnancy, after delivery, among newborns and children from 48 to 70% [9]. Beneficial effects of mHealth studies are also available from the field of maternal and child health [10], malaria [11, 12], diabetes [13, 14], HIV/AIDS [15], sexual and reproductive health [16], health behaviour change [17] etc.

Against this background, ReMiND (reducing maternal and newborn deaths) program was introduced in two blocks of district Kaushambi in state of Uttar Pradesh. As part of this program a mHealth application which runs on an open source platform was introduced as job aid for ASHA workers. This mHealth application tracks and supports clients for the ASHA workers and provides inputs for individualized service and counselling needs [18, 19]. ReMiND intervention resulted in a statistically significant increase in coverage for iron and folic acid (IFA) tablets consumption among pregnant women (12.7%), abdominal examination during ANC (18.7%), identification and self-reporting of complication during pregnancy (13.20%) and after (19.5%) delivery in the intervention area [20].

The need for optimal utilization of available resources with the help empirical data gains importance in resource constrained country like India. Realising this National Health Policy 2017 emphasised on the use of Health Technology Assessments (HTA) as a tool for taking informed decisions in scaling up health interventions in India [21]. The use of HTA is also supported by Disease Control Priority (DCP 3) and experts across globe for getting maximum health benefits out of available pool of resources [22–24]. However, a systematic review of economic evaluations of mHealth interventions concluded that there is a lack of concrete evidence to fully assess the economic impact of telemedicine, e-health, and mHealth systems [25]. Deficiencies in design of studies, such as lack of randomized control trials, small sample sizes, and absence of quality data and appropriate measures further limit the relevance of findings. Furthermore, though effectiveness studies from low- and middle-income countries are available in literature, there is no evidence on cost effectiveness of mHealth interventions from India [26]. In this paper, we assessed the incremental cost per disability adjusted life year (DALY) averted as a result of the ReMiND intervention as compared to routine care without the mHealth intervention in Uttar Pradesh state in India.

## Methods

### Intervention setting: ReMiND intervention and theory of change

The intervention scenario comprised of routine maternal and child health care services plus the ReMiND intervention. Out of Kaushambi district’s eight community development blocks, the ReMiND was implemented in two blocks: Mooratganj and Manjhanpur. In 2012, mHealth application was implemented through 259 ASHAs in two intervention blocks serving a population of about 300,000 individuals. The ASHAs in ReMiND program were provided with basic Java-based mobile phones operating on an open-source Comm-Care software [19]. It had a tailored content, which guided the ASHA through the course of a woman’s pregnancy and newborn child care. More specifically, it was used to register the pregnant woman; update her ANC record during subsequent home visits; track her utilization of services from pregnancy into the postpartum period; and track the health of the newborn and the immunization until 2 years of age. They were given extensive trainings on use of mobile phone which also provided audio-visual support to the ASHA workers in order to counsel the pregnant woman at each of these steps [18, 19].

Data entered about the pregnancy guided ASHAs in providing timely and appropriate health information to pregnant women; and helped them to prioritize home visits. It contained algorithms to assist in the early identification, treatment, and rapid referral for appropriate care of any danger signs among pregnant women or neonates [18, 19]. Data on services which are due and those utilized by pregnant women, recorded by ASHAs through the mHealth application, were pooled on a common server. The sector facilitators used the data to monitor all the ASHAs working in their area. Data were also shared with the health education officer at the primary health centre during monthly meetings. Thus, the implementing NGO partners—Catholic Relief Services (CRS) and Vatsalya worked in coordination with the district health system to monitor the performance of ASHAs using data generated by the mHealth application.

The purpose of the mHealth application was to improve the quality of counselling by ASHA worker, which in turn was aimed to improve the knowledge of pregnant women. Ultimately, this was intended to generate demand for seeking antenatal and natal services; and for timely care of complications during pregnancy, after delivery and during neonatal period [19]. The increased utilization of preventive health services, as a result of demand generation and better supply-side monitoring, is likely to result in lower illnesses and as a result reduction in mortality and disability. Similarly, improved care-seeking can also bring about a reduction in fraction of illnesses which are fatal or which result in long-term complications.

### Counterfactual: routine care

The routine care scenario comprised of delivery of preventive and curative maternal and child health services, including implementation of the flagship program—National Health Mission [27]. These comprised of all the set of basic demand and supply side services which are recommended for maternal, newborn, child and adolescent health care as envisaged under the Reproductive, Maternal, Newborn, Child and Adolescent Health Care program (RMNCHA+) [28]. It was launched in year 2013 to provide a continuum of care right from the start of reproductive age of a girl child to the adolescent health of her offspring. It envisages health system strengthening for providing antenatal care, intra-partum, postpartum care to women for safe maternity, essential new born, early identification and referral services in case of any complication, immunization, prevention and treatment of childhood morbidities, family planning along with interventions for improving physical and psychological health in adolescents. The only difference between the intervention and the control area was the rollout of mHealth application which was used by ASHA workers.

### General model overview

A decision tree (Additional file 1: Figure S1) was parameterized on MS-Excel spreadsheet to estimate the incremental cost effectiveness of implementing ReMiND. A time horizon of 10 years starting from base year of 2011 was considered appropriate to cover all costs and effects comprehensively on grounds of intervention characteristics and theory of change for effectiveness mediation. This time horizon was justified based on several reasons. Firstly, the m-health software is unlikely to change in this period as the broad nature of services will remain same. Secondly, based on expert opinion, even if the software has to be edited based on revisions in the program package, such changes are unlikely to have any major cost implications. Thirdly, while several costs of capital nature are incurred during the early years of implementation, however, the consequences of those investments i.e. health benefits continue to occur till many years later. These health effects are likely to occur during pregnancy (such as reduction of high-risk pregnancies, and their early detection and appropriate management), childbirth (such as reduction in post-partum haemorrhage), neonatal (reduction of low-birth weight, and prevention and management of neonatal illnesses), infancy and childhood period (such as prevention of vaccine preventable diseases) up to 5–10 years of age. Finally, economic evaluations of similar m-health packages have also relied on a similar 10–12 year time horizon [29, 30].

We analyzed costs and effects from both health system and societal perspective. Health system costs included the resources spent by the department of health and the implementing partners in delivering the intervention. These included resources such as building, space, staff salaries, equipment, software for m-health intervention, medicines, consumables, overheads etc. While measuring the societal costs, in addition to the health system cost, we also measured the out-of-pocket expenditures (OOPE) incurred by households. These OOPE were incurred for purchasing medicines, medical or surgical procedures, boarding, lodging, and transportation as a result of any health care sought during the pregnancy, intra-partum care, or neonatal period. We did not include the measurement of indirect costs in terms of productivity loss to the household as a result of absenteeism due to illness. Effect was measured in terms of illness episodes averted, maternal and neonatal deaths prevented, life years gained and DALYs averted. Both costs and effects were discounted at 3% to account for time preference of cost and utility. The choice of discount rate is justified on the following grounds. First, as per World Health Organization’s Choosing Health Interventions for Cost Effectiveness guidelines (WHO-CHOICE), it has been recommended to discount all future costs and consequences at 3% for international comparability [31]. This is in coherence with the recent guidelines released by Disease Control Priority 3 [22] and the reference case developed for low middle income countries by International Development Support Initiative and the Gates Foundation [32]. Also a recent systematic review of the economic evaluations done in India revealed that 82% of the studies which reported the value of discount rate, 3% rate was used to discount future costs and benefits [26]. To account for uncertainty in value of discount rate, we varied it up to 8% in probabilistic sensitivity analysis. The concept of discounting incorporates both the components i.e. time preference and inflation rate. Time preference represents the opportunity cost of an investment.

We report our findings as incremental cost of implementing ReMiND intervention per DALY averted, per illness episode prevented and per infant death averted as compared to routine care services [33]. An incremental cost-effectiveness ratio (ICER) is a summary measure in economic evaluations to represent the economic value of an intervention in comparison with an alternative or no alternative (comparator). The ICER is expressed as the ratio of the difference in costs between two strategies to the difference in effectiveness. For preference based outcomes, disability rates were taken from the Global Burden of Disease data [34, 35].

There are several thresholds which could be used for decision making in a cost-effectiveness analysis [36]. It could be a supply-side threshold, demand-side threshold or GDP based thresholds. Supply-side threshold is a measure of health benefits forgone due to reduced funding for current interventions as a result of allocating resources for a new intervention from provider’s perspective. A demand-side threshold describes the willingness to pay of an individual to gain additional health benefits in view of other competing demand of his resources. Third, the per capita GDP of a country recommended by several guidelines in the absence of evidence on other threshold measures [31]. The approach suggested by the commission for Macroeconomics on Health (2001) is that interventions with an incremental cost per DALY averted less than the per capita GDP in low middle income countries (LMICs) are “very cost effective”, and those costing less than triple the per capita GDP are “cost- effective”. In India, till date, there is a scarcity of evidence on supply-side and demand-side thresholds. Hence, per capita GDP is the most commonly used threshold in economic evaluations done in India [37–39].

The standard guidelines for conducting and reporting an economic evaluation survey (CHEERS) were adhered to and details are available as Additional file 2: Appendix S1.

### Costing

We analysed the costs from both health system and societal perspective. The health system costs comprised of four distinct components—firstly, it included the cost of implementing the mHealth application, i.e. development of software, training of ASHA workers, mobile phones and data transmission charges etc. [40]. These costs were obtained in US dollars which were converted into Indian rupees using dollar exchange rates given by Internal Revenue Service for year 2015 (1US$ = INR 63.35) [41]. The converted rates were then inflated from the year of purchase to the current value of product in 2015 by applying Consumer Price Index in India [42]. Secondly, we considered the incremental time spent for monitoring and supervision of this additional activity by implementing partners and state health department. Thirdly, introduction of intervention could have resulted in change in the allocation of time for provision of services by ASHA workers and hence affecting the staff costs. However, ASHA workers are not full-time paid staff, and are instead paid a performance-based incentive. As a result, determining the time allocation was not meaningful in this scenario. Instead, we estimated the total performance based payment paid to ASHA workers in intervention and control area to compute the difference. The mHealth application was intended to increase the counseling skills of ASHAs and better understanding of the beneficiaries; we found that there was no significant increase in the utilization of incentive based services which largely includes institutional deliveries. Hence, the incremental costs related to ASHAs incentive were not included in the analysis.

Finally, the intervention could have brought about change in utilization of heath care services, which entails a cost. The benefits reaped as a result of improved knowledge and treatment-seeking in the intervention population were inherent in overall benefits measurement and therefore, we included its associated increase in cost (i.e. cost of increased utilization of healthcare services). As a result, we estimated the cost of delivering extra services—preventive or curative. We used the unit cost of providing mHealth intervention under ReMiND in two blocks of Kaushambi as estimated in the cost analysis—i.e. INR 31.4 (US $ 0.49) per capita and INR 1294 (US $ 20.5) per pregnant woman [40]. The cost per pregnant woman is most appropriate which incorporate not only population distribution but also fertility levels and thus, allows modeling of costs in a scale up scenario in most appropriate way. The cost per woman of reproductive age captures the age distribution of fertile women in the population but it does not capture the level of fertility and its effect on cost of program implementation. The unit cost capita is also inappropriate for use in the economic evaluation, as it neither captures the effect of population demographics, nor fertility.

The cost of delivering preventive and curative health services at different levels of the health care delivery system in another study from North Indian states was used [43, 44]. The preventive services included in our analysis were maternal and child health services like provisioning of antenatal care (consumption of iron folic acid tablets, tetanus toxoid vaccine, number of ANC visits), postnatal care, essential newborn care and full immunization till 1 year of age. The curative services included institutional deliveries; treatment of complications during pregnancy, after delivery, among newborn and infants in either outpatient or inpatient setting at various levels of health care facilities. These studies had employed bottom up costing methods to comprehensively estimate the cost of delivering services in a representative sample of sub-centers, primary health centre, community health centre and district hospitals. Unit costs for antenatal care, postnatal care, and immunization were INR 525 (USD 10) per full ANC care, INR 767 (USD 14) per PNC case registered, and INR 97 (USD 1.8) per child immunized in routine immunization respectively [43]. Similarly, the cost incurred on per outpatient consultation at PHC and CHC was taken as INR 120 (95% CI 90–151) and 126 (95% CI 92–160) respectively while the unit cost per hospitalization was INR 1156 (95% CI 343–2140) at PHC and INR 1115 (95% CI 400–2188) at CHC level [45]. The cost per OPD consultation and bed day hospitalization for gynaecology (INR 165; 997) and paediatrics (INR 137; 1028) department at district hospital respectively were taken for our model [46]. The detailed cost analysis is provided in Additional file 3: Appendix S2.

In order to assess the change in utilization of health care services, we analysed the care seeking behaviour of pregnant women for illnesses/complications during delivery and after child-birth. This was assessed based on analysis of a household survey—CEAHH (cost effectiveness analysis household survey) survey, which was used to determine care seeking for illnesses reported in pregnancy, after child-birth and during neonatal period [18]. The out of pocket expenditure estimates for seeking outpatient and inpatient care for various maternal and childhood illnesses were given in Table 1. Expenditures by households’ in the form of OOPE were included along with health system costs to estimate cost to the society from a societal perspective. Details for the household survey are available elsewhere in the protocol and impact assessment papers [18, 20].

**Table 1:** Demographic and epidemiological parameters

Parameter (base year: 2011)	Base value	Lower limit	Upper limit	Distribution	Source
Demographic parameters					
Total population of Uttar Pradesh State	199,812,341	169,840,490	229,784,192	Lognormal	Census 2011
Birth rate (per 1000 population)	27.8	27.5	29.1	Lognormal	Census 2011
Annual decline in birth rate (%)	− 0.04	− 0.03	− 0.05	Lognormal	
Maternal mortality ratio (per 100,000 live births)	258	241	275	Lognormal	Annual Health Survey (AHS) Report, 2012–2013
Neonatal mortality rate (per 1000 live births)	40	23	43	Lognormal	Census 2011
Epidemiological parameters					
Prevalence of anaemia in pregnant women	0.51	0.34	0.68	Beta	NFHS-4, 2015–2016
Risk of anaemia: for women taking IFA during pregnancy	0.25	0.21	0.28	Beta	Haider et al. [47]
Risk of anaemia: for women not taking IFA during pregnancy	0.75	0.60	0.90	Beta	
Risk of postpartum haemorrhage (PPH) among anaemic pregnant women	0.29	0.19	0.39	Beta	Prata et al. [48]
Risk of prematurity among anaemic pregnant women	0.63	0.33	1.01	Beta	Rahman et al. [49]
Risk of low birth weight (LBW) among anaemic pregnant women	0.31	0.13	0.51	Beta	
Probability of maternal mortality with PPH: with treatment	0.00038	0.00029	0.00047	Beta	Prata et al. [48]
Probability of maternal mortality with PPH: without treatment	0.00051	0.00044	0.00058	Beta	
Probability of neonatal mortality due to prematurity: with treatment	0.102	0.082	0.122	Beta	Bang et al. [50]
Probability of neonatal mortality due to prematurity: without treatment	0.332	0.266	0.398	Beta	
Probability of neonatal mortality due to LBW: with treatment	0.047	0.038	0.056	Beta	
Probability of neonatal mortality due to LBW: without treatment	0.113	0.090	0.136	Beta	
Prevalence of hypertension (HTN) in pregnancy	0.07	0.047	0.093	Beta	NFHS-4, 2015–2016
Risk of preeclampsia in hypertensive pregnant women	0.63	0.422	0.838	Beta	Borade et al. [51]
Risk of eclampsia in preeclampsia pregnant women	0.115	0.077	0.153	Beta	
Risk of perinatal complications due to eclampsia	0.524	0.351	0.697	Beta	The Magpie Trial 2007
Probability of maternal mortality due to eclampsia among pregnant women: with treatment	0.18	0.144	0.216	Beta	
Probability of maternal mortality due to eclampsia among pregnant women: without treatment	0.4	0.32	0.48	Beta	
Probability of neonatal mortality due to perinatal complications: with treatment	0.102	0.082	0.122	Beta	Bang et al. [50]
Probability of neonatal mortality due to perinatal complications: without treatment	0.332	0.266	0.398	Beta	
Risk of maternal mortality in home deliveries	0.02	0.016	0.024	Beta	Montgomery et al. [52]
Risk of maternal mortality in institutional deliveries	0.00279	0.00223	0.00335	Beta	
Prevalence of sepsis in neonates	0.03	0.02	0.04	Beta	National Neonatal Perinatal Database
Probability of neonatal deaths due to sepsis: with treatment	0.18	0.14	0.22	Beta	Seale et al. [53]
Probability of neonatal deaths due to sepsis: without treatment	0.95	0.95	0.95	Beta	
Average length of illness (years): anemia	0.75	0.60	0.90	Lognormal	Expert opinion
Average length of illness (years): PPH	0.01	0.01	0.01	Lognormal	
Average length of illness (years): HTN/eclampsia	0.75	0.60	0.90	Lognormal	
Average length of illness (years): prematurity	0.03	0.02	0.03	Lognormal	
Average length of illness (years): LBW	0.03	0.02	0.03	Lognormal	
Average length of illness (years): sepsis	0.03	0.02	0.03	Lognormal	
Average length of illness (years): perinatal complications	0.03	0.02	0.03	Lognormal	
Impact parameters					
Increase in coverage of 3 ANC visits (%)	0.00	0.00	0.00	Lognormal	ReMiND-impact assessment study
Increase in coverage of IFA (%)	12.70	8.70	16.70	Lognormal	
Increase in coverage of TT (%)	0.00	0.00	0.00	Lognormal	
Increase in coverage of care seeking (%)	25.7	13.70	41.10	Lognormal	
Increase in coverage of institutional delivery (%)	0.00	0.00	0.00	Lognormal	
Cost parameters (INR)					
Health system costs					
Unit cost: ANC	525	456	619	Gamma	Prinja et al. [54]
Unit cost: PNC	767	538	1092	Gamma	
Unit cost: immunization	97	77	120	Gamma	
Unit cost: institutional delivery	1872	1080	2990	Gamma	Prinja et al. [37] (PLOS one)
Unit cost: PHC					
OPD	120	90	151	Gamma	
IPD	1156	343	2140	Gamma	
Unit cost: CHC					
OPD	126	92	160	Gamma	
IPD	1115	400	2188	Gamma	
Unit cost: gynaecology and obstetrics					
OPD	165	68	274	Gamma	Prinja et al. [55] (IJMR)
IPD	997	592	1412	Gamma	
Unit cost: paediatrics					
OPD	137	102	182	Gamma	Prinja et al. [55] (IJMR)
IPD	1028	444	1703	Gamma	
Out of pocket expenditures (INR)					
Control: public					
ANC	406	305	508	Gamma	Primary data analysis (CEAAH)
Institutional delivery	610	548	672	Gamma	
Postpartum care	1216	912	1520	Gamma	
Neonatal illness					
OPD	283	29	601	Gamma	
IPD	2357	852	6908	Gamma	
Control: private					
ANC	845	634	1056	Gamma	
Institutional delivery	13,000	11,154	14,846	Gamma	
Postpartum care	699	524	874	Gamma	
Neonatal illness					
OPD	769	472	1066	Gamma	
IPD	5164	3039	7289	Gamma	
Intervention: public					
ANC	878	659	1098	Gamma	
Institutional delivery	861	713	1009	Gamma	
Postpartum care	450	338	563	Gamma	
Neonatal illness					
OPD	380	145	615	Gamma	
IPD	1399	1049	1749	Gamma	
Intervention: private					
ANC	1420	1065	1775	Gamma	
Institutional delivery	16,900	11,051	22,749	Gamma	
Postpartum care	1791	1343	2239	Gamma	
Neonatal illness					
OPD	800	500	1100	Gamma	
IPD	1000	750	1250	Gamma	

### Valuing consequences of ReMiND intervention

ReMiND intervention was intended to improve the quality of counselling of pregnant woman by the ASHA worker. Improved counselling was desired to improve knowledge of pregnant women, and utilization of appropriate maternal and child health care services, during pregnancy, child-birth and during the neonatal period. Secondly, the data entered by the ASHA worker helped in tracking the ASHA worker to track pregnant women and their services utilized; besides being used for supervision of ASHA performance [18].

We undertook a pre and post quasi experimental study to assess the impact of the intervention. Two blocks other than two intervention blocks were selected as controls after matching for coverage of two indicators at baseline—ante natal care and institutional deliveries from the same district. The pre-intervention data was obtained from the Annual Health Survey 2011 conducted by Ministry of Health and Family Welfare. A household survey was carried out in four blocks of Kaushambi district in year 2015 to observe the post intervention coverage. Propensity score matched sample from intervention and control areas in pre-intervention and post-intervention periods were analysed using difference-in-difference method to estimate the impact of ReMiND program. Overall, the ReMiND led to a statistically significant increase in coverage for IFA consumption (12.7%), abdominal examination during ANC care (18.7%), identification and self-reporting of complication during pregnancy (13.20%) and after (19.5%) delivery and care seeking (25.7%) in the intervention area [20, 56]. The coverage of three or more ANC visits, tetanus toxoid vaccination, full ANC care and ambulance usage also increased in intervention area by 10.3, 4.3, 1 and 2.5%; however, the difference between the improvements in the intervention and control area was not statistically significant [20] (Table 1).

The coverage of MNCH services in control area from baseline and end-line surveys was used to interpolate the coverage during intervening years, and extrapolate during the future years from 2015 onwards. Linear change was assumed for the purpose of modelling. In case the coverage for any indicator reached 90%, no further increase was assumed thereafter in the subsequent years. Similarly, the impact estimates of difference in difference for intervention area were used to compute annual rate of change in the intervention area, which was further used to model the coverage of MNCH services in intervention scenario, relative to the counterfactual.

In this paper, we model the effect of increased utilization of health care services on reduction in illnesses or complications during pregnancy and after child-birth. Together, these two contributed to reduction in maternal and neonatal deaths—ultimately resulting in averting years of life lost (YLL) to premature mortality and reduction of disability adjusted life years (DALY). In terms of maternal complications, we primarily modelled the effect of changes in antenatal services on two major illnesses during pregnancy—anaemia and hypertension. These two were particularly considered in view of their prevalence in the targeted population [57], as well as evidence linking reduction in occurrence of these medical conditions with better ANC care [58]. Baseline prevalence of 51.4 and 5.8% was assumed for anaemia and hypertension during pregnancy [59]. Subsequently, we modelled the effect of improvement in coverage of complete IFA supplementation as a result of mHealth intervention—risk of anaemia is 75 and 25% without and with IFA supplementation respectively [47]. Similarly, an 11.5% risk of developing eclampsia was assumed among hypertensive pregnant women [51]. In turn, we assumed that anaemia results in complications during pregnancy and after child-birth, such as post-partum haemorrhage (29%), and is also associated with adverse neonatal health outcomes such as prematurity (63%) and low birth weight (31%) [49]. Finally, reduction of post-partum haemorrhage is associated with reduced maternal mortality [60]; while both prematurity and low birth weight results in a higher risk of neonatal mortality [50] (Table 1).

Secondly, the ReMiND intervention resulted in improved recognition of the danger signs during pregnancy and after child birth. The care seeking for any illness during pregnancy was higher in the intervention area (71.9%) as compared to control are (46.2%). We modelled the impact of improved care seeking on maternal and neonatal survival. For example, the risk of maternal mortality with post-partum haemorrhage is 25% less with treatment than without [60, 48].

Besides an increase in YLL as a result of reduction in mortality, we also estimated the reduction in years of life lived in disability (YLD) as a result of reduced illnesses during pregnancy, after child-birth and during neonatal period. We used the disability weights as provided in the Global Burden of Disease, 2010, for computing YLD [61]. For calculating YLL in case of an infant death, we estimated that the mean age of infant death is 26 days. This estimation was based on the assumption that 60% of infant deaths occur in neonatal period, 60% of neonatal deaths are early neonatal deaths (within first 7 days of birth) [62–64]. We also assumed that mean age of early neonatal, late neonatal and post-neonatal death is 3, 20 days and 6 months respectively; i.e. the mid-point of class interval. Similar assumptions have also been used by another study evaluating cost effectiveness of IMNCI program in India [37]. We computed the percentage reduction in maternal and neonatal deaths by comparing the number of deaths in intervention where ReMiND program was implemented relative to control using a decision tree model (Additional file 1: Figure S1). Table 1 cites the estimates of effectiveness on proximal outputs i.e. coverage of services, and assumptions for modeling impact on long term outcomes such as morbidity and mortality derived from the Indian studies.

### Sensitivity analysis

We undertook a probabilistic sensitivity analysis to test the effect of parameter uncertainty on the findings of the analysis and to estimate the effect of joint uncertainty in all parameters [65–67]. While base analysis is valid for UP state, there is significant variability in values for various parameters from Indian perspective which was important to test in the PSA analysis. For several parameters related to unit cost of health care services, effectiveness estimates of mHealth interventions, some demographic parameters, and service coverage in counterfactual scenario etc., 95% confidence intervals were available from primary analysis as part of the current study or secondary literature [40, 44, 45, 54, 55, 68]. For other parameters, such as demographics and epidemiological parameters such as risk of various morbidities with or without use of preventive interventions etc., we varied the base estimate obtained from literature 20% on either side. For certain parameters, such as risk of mortality with and without treatment we varied the base estimate by 50% on either side, since this is heavily dependent on other supply side inputs which could vary significantly across different parts of the country. In case of prevalence of risk factors such as anaemia and hypertension during pregnancy, or low birth weight babies, we varied the base parameter by 33% on either side. Finally, we varied the cost of mHealth intervention by 50% on lower side and 20% on higher side. The same was done as we expect that implementation of mHealth intervention through support from a donor partner would be higher than when it is implemented through public sector health system which has relatively lower salary structures.

Probability of ReMiND intervention to remain cost effective at a willingness to pay threshold equal to per capita gross domestic product (GDP) was estimated, using a health system perspective. For undertaking PSA analysis, we assumed a lognormal distribution for unit costs. In case of parameters where 95% confidence interval was available, a beta distribution was used; while uniform distribution was applied where an upper and lower bound were available. Monte Carlo method was used for simulating the results 999 times. Median was computed along with 2.5th and 97.5th percentile to estimate 95% confidence interval.

## Results

### Costs

**Fig. 1:**
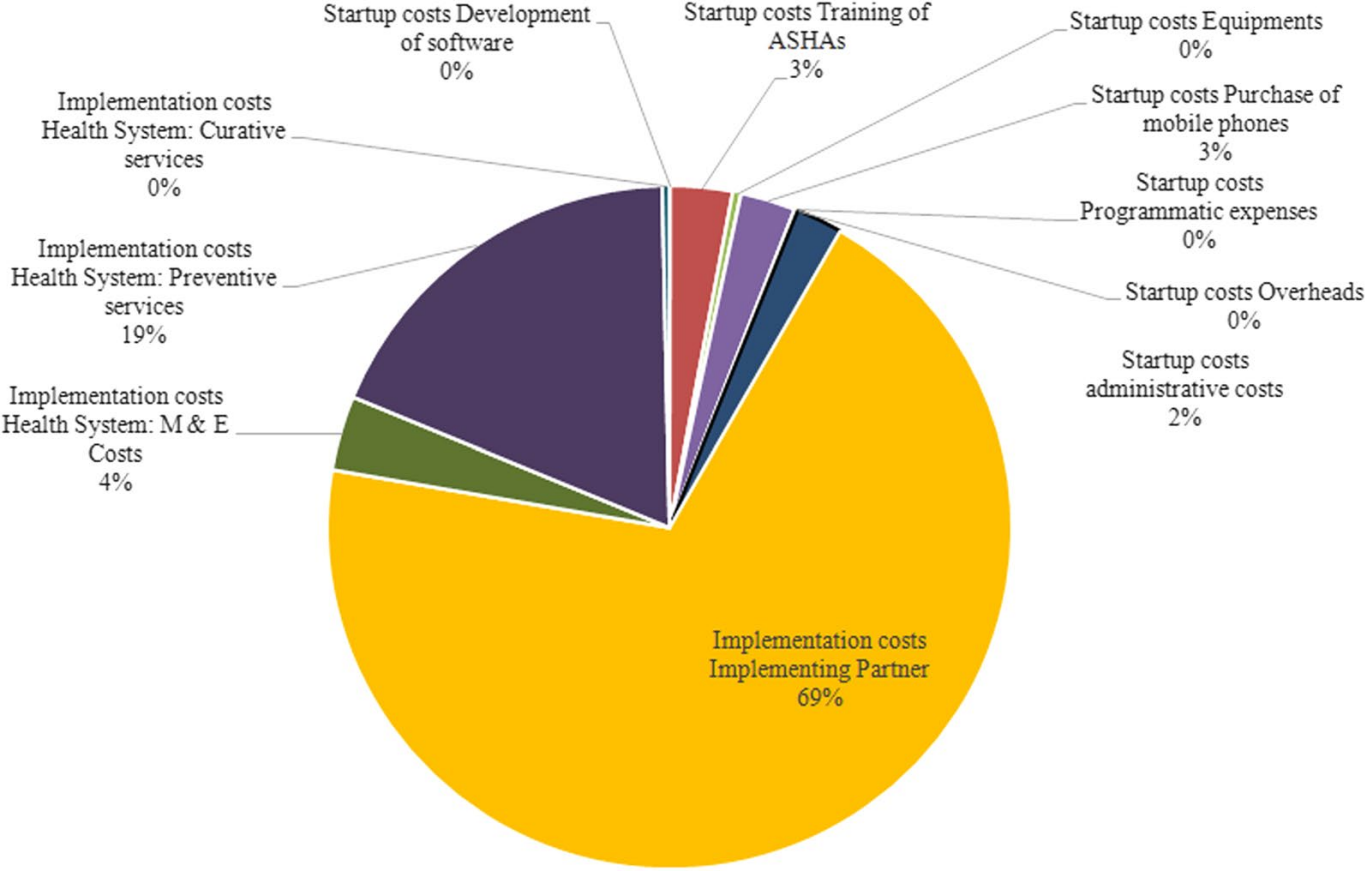
Distribution of incremental costs of mHealth intervention: start up and implementation cost

**Fig. 2:**
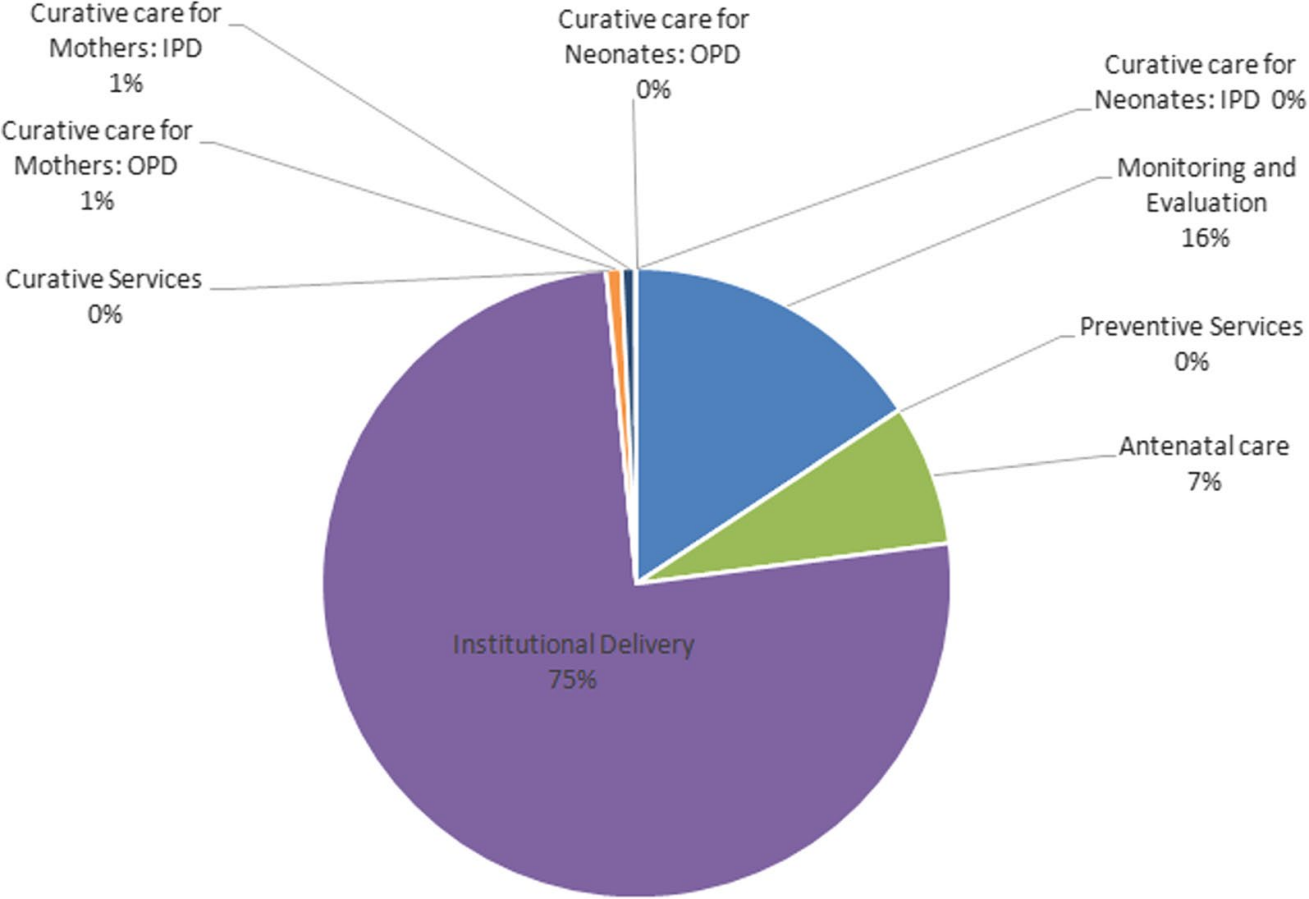
Distribution of incremental health system costs

Overall, we found that the implementation of ReMiND in UP state from 2011 to 2020 would save 4,127,529 DALYs at an incremental cost of USD 982 million (Tables 2, 3). More than 90% of this cost is on account of implementation of the intervention which includes monitoring and supervision of the intervention, cost of increased uptake of preventive services such as ANC care and institutional deliveries; and curative services [40]. Implementing partners’ incurred almost 3/4th share of the implementation cost (Fig. 1). Interestingly, the cost of curative care in the intervention scenario is less than the counterfactual. This could be a result of reduction in illnesses during pregnancy and after child-birth as a result of increase in preventive interventions. This reduction in illnesses was significant enough to offset the inflationary effect of improved care seeking on the health system costs. In terms of the start-up costs, which constituted about 10% of incremental cost of intervention, little over one-third (37%) comprised of the resources spent for training the ASHA workers [40]. Health system spent majority of its costs on institutional deliveries (75%) followed by monitoring and supervision (16%) (Fig. 2). From societal perspective, there was a cost saving of USD 425 million with ReMiND intervention (Table 2). These cost savings were mainly due to two reasons. First, with ReMiND intervention, there was an increased uptake of preventive services like ANC which led to reduction in number of maternal and neonatal illnesses in the intervention scenario and therefore, decreased demand for curative care. Second, with better contact with public health system, more people utilized public health facilities both for preventive and curative care and hence, incurred less amount of OOPE (Tables 1 and 2).

**Table 2:** Incremental costs (in INR and USD) of ReMiND intervention in Uttar Pradesh state, India

Incremental costs of m-health program (2015)	Base value		Lower limit		Upper limit	
	INR	USD	INR	USD	INR	USD
Startup costs (mHealth)						
Development of software	17,495,229	276,168	8,747,615	138,084	20,994,275	331,401
Training of ASHAs	1,826,036,757	28,824,574	913,018,378	14,412,287	2,191,244,108	34,589,489
Equipments	244,654,336	3,861,947	122,327,168	1,930,973	293,585,203	4,634,336
Purchase of mobile phones	1,618,284,035	25,545,131	809,142,018	12,772,565	1,941,940,842	30,654,157
Programmatic expenses	98,490,876	1,554,710	49,245,438	777,355	118,189,051	1,865,652
Overheads	199,921	3156	99,960	1578	239,905	3787
Administrative costs	1,361,328,050	21,488,998	680,664,025	10,744,499	1,633,593,660	25,786,798
Total	5,166,489,205	81,554,684	2,583,244,602	40,777,342	6,199,787,046	97,865,620
Implementation costs (mHealth)	43,130,264,882	680,825,018	21,565,132,441	340,412,509	51,756,317,858	816,990,021
Health system incremental costs (with mHealth)						
Monitoring and evaluation	2,178,513,501	34,388,532	1,089,256,751	17,194,266	2,614,216,201	41,266,238
Preventive services						
Antenatal care	1,000,908,828	15,799,666	869,360,811	13,723,138	1,180,119,171	18,628,558
Institutional delivery	10,462,218,682	165,149,466	6,035,895,393	95,278,538	16,710,488,173	263,780,397
Curative services						
Curative care for mothers: OPD	109,167,717	1,723,247	52,210,647	824,162	272,919,293	4,308,118
Curative care for mothers: IPD	97,857,094	1,544,706	54,078,920	853,653	178,899,733	2,823,989
Curative care for neonates: OPD	3,828,183	60,429	2,734,416	43,164	30,156,706	476,033
Curative care for neonates: IPD	3,655,308	57,700	643,294	10,155	11,305,025	178,453
Total (health system)	62,152,903,400	981,103,448	32,252,557,276	509,116,926	78,954,209,206	1,246,317,430
Incremental out of pocket expenditures (with mHealth)						
ANC	− 1,033,319,209	− 16,311,274	− 1,345,597,054	− 21,240,680	− 697,526,728	− 11,010,682
Institutional delivery	− 89,638,635,385	− 1,414,974,513	− 116,728,192,568	− 1,842,591,832	− 60,509,224,497	− 955,157,451
Postpartum care	1,392,533,772	21,981,591	1,813,369,309	28,624,614	940,009,163	14,838,345
Pediatrics: OPD	203,844,023	3,217,743	265,447,419	4,190,172	137,601,869	2,172,089
Pediatrics: IPD	9,438,499	148,990	12,290,894	194,016	6,371,318	100,573
Total (out of pocket expenditures)	− 89,066,138,301	− 1,405,937,463	− 115,982,682,000	− 1,830,823,710	− 60,122,768,874	− 949,057,125
Grand total	− 26,913,234,901	− 424,834,016	− 59,581,747,680	− 940,516,933	76,787,545,236	1,212,115,947

*INR* Indian National Rupee, *USD* United States Dollar

**Table 3:** Effectiveness and cost effectiveness of m-health program in Uttar Pradesh state, India (2011–2020)

Characteristics	Base case	Lower limit	Upper limit
Health outcomes with routine services			
Maternal illness episodes	34,598,786	21,796,486	50,063,694
Neonatal illness episodes	1,941,237	1,292,080	2,725,595
Maternal deaths	96,921	79,833	117,305
Neonatal deaths	2,168,635	1,833,492	2,548,800
Life years lost	32,835,472	19,235,215	61,273,469
DALYs lost	53,028,454	32,271,008	89,078,621
Health outcomes with routine health services and m-health			
Maternal illness episodes	31,444,322	18,681,922	46,116,428
Neonatal illness episodes	1,903,900	1,270,809	2,677,032
Maternal deaths	96,609	79,688	116,916
Neonatal deaths	2,019,167	1,700,694	2,389,956
Life years lost	30,604,197	17,896,762	57,353,515
DALYs lost	48,900,926	29,160,101	83,178,589
Incremental benefits with m-health			
Maternal illness episodes averted	− 3,154,464	− 3,114,564	− 3,947,266
Neonatal illness episodes averted	− 37,337	− 21,271	− 48,564
Maternal deaths averted	− 312	− 146	− 389
Neonatal deaths averted	− 149,468	− 132,798	− 158,844
Life years saved	− 2,231,275	− 1,338,453	− 3,919,955
DALYs averted	− 4,127,529	− 3,110,907	− 5,900,032
Incremental cost effectiveness ratio, health system perspective			
Cost per illness averted in INR (USD)	15,208 (240)	12,372 (195)	18,958 (299)
Cost per death averted in INR (USD)	371,577 (5865)	253,502 (4002)	526,418 (8310)
Cost per life year gained in INR (USD)	25,371 (400)	13,461 (212)	47,428 (749)
Cost per DALY averted in INR (USD)	12,993 (205)	8570 (135)	18,523 (292)
Incremental cost effectiveness ratio, societal			
Cost per illness averted in INR (USD)	− 6656 (− 105)	− 39,965 (− 631)	2608 (41)
Cost per death averted in INR (USD)	− 162,634 (− 2567)	− 1,109,743 (− 17,518)	53,433 (843)
Cost per life year gained in INR (USD)	− 11,105 (− 175)	− 99,982 (− 1578)	2837 (45)
Cost per DALY averted in INR (USD)	− 5687 (− 90)	− 39,049 (− 616)	1806 (29)

*INR* Indian National Rupee, *USD* United States Dollar

### Valuation of consequences

**Fig. 3:**
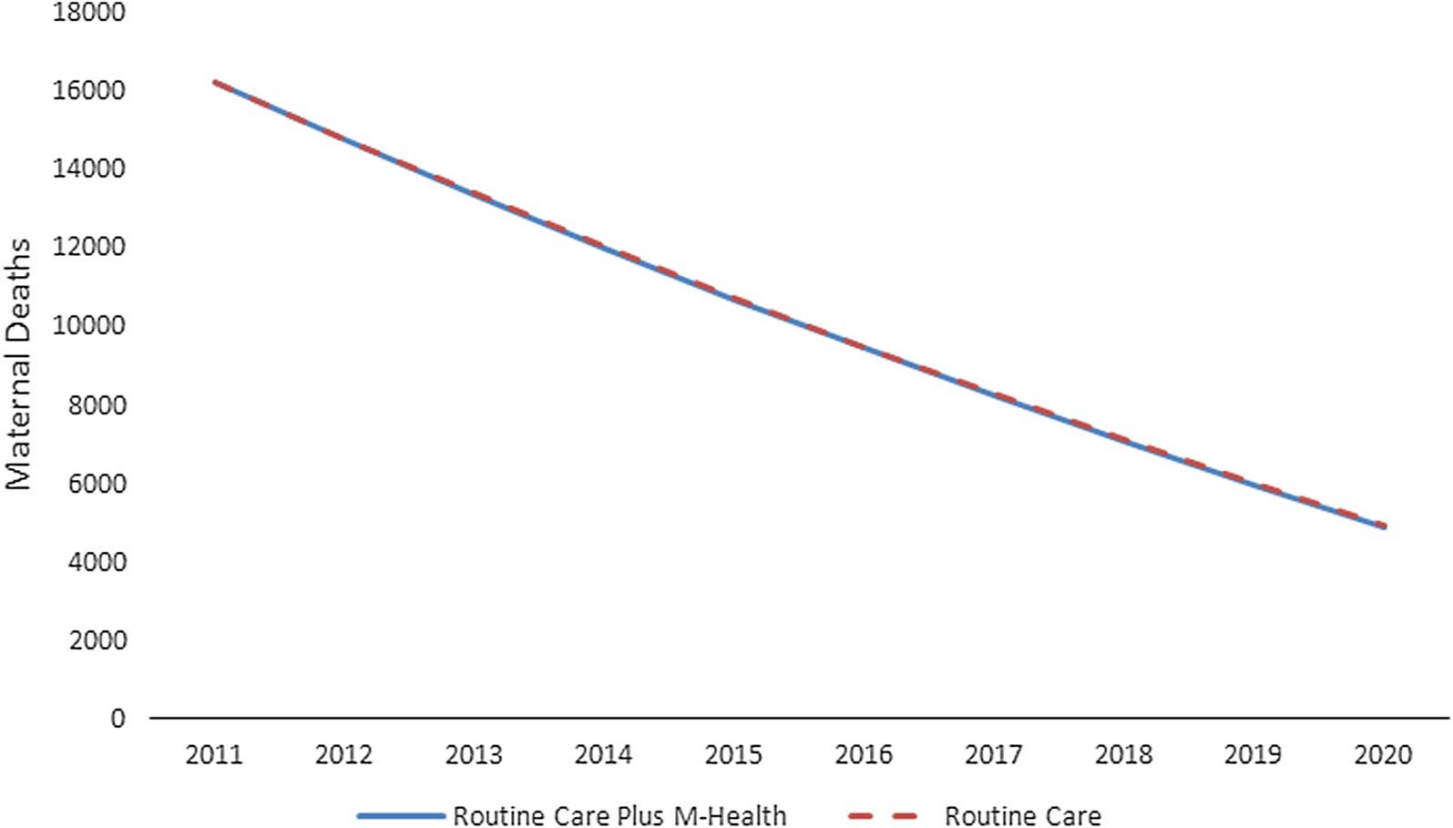
Trend of maternal deaths in Uttar Pradesh, 2011–2020

**Fig. 4:**
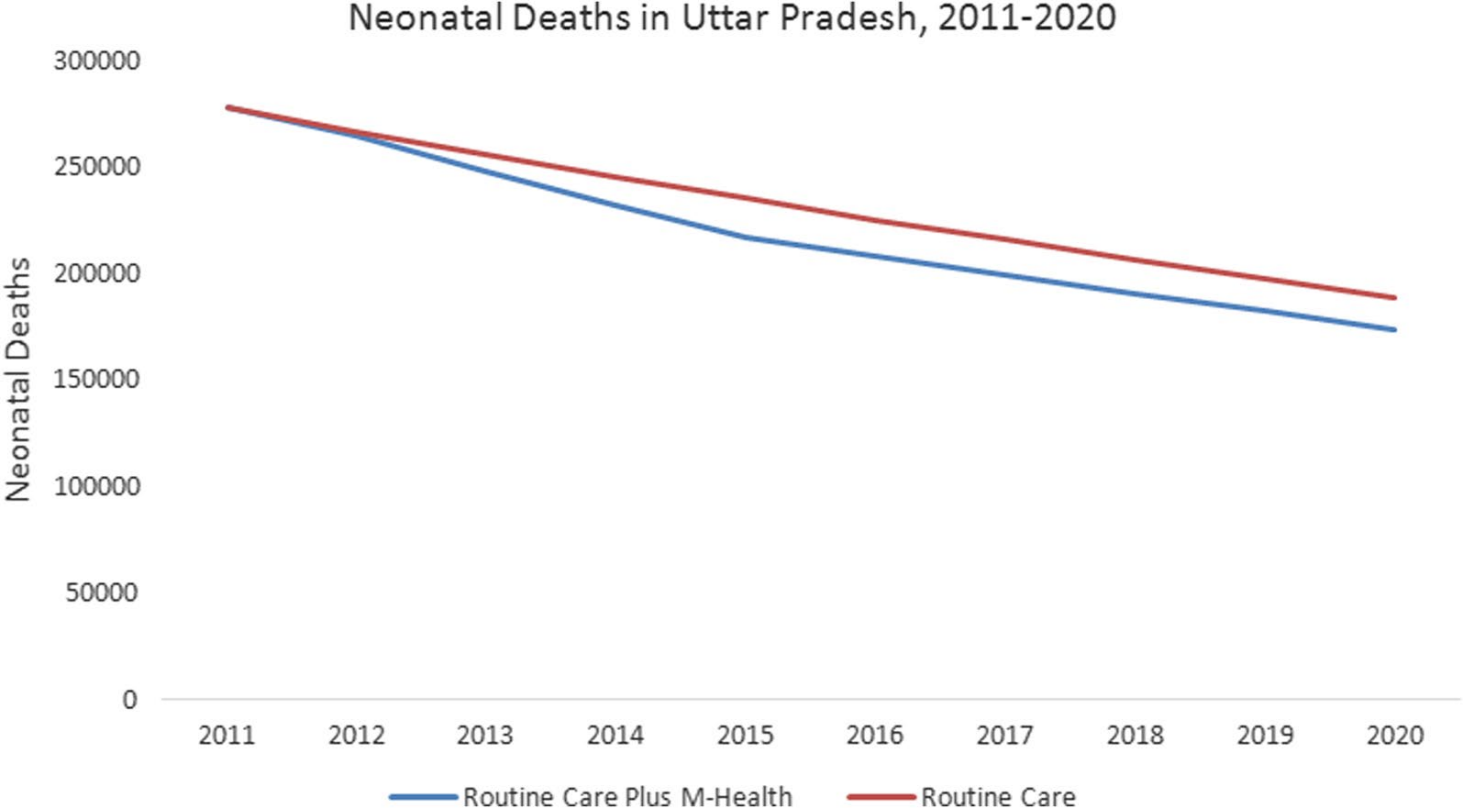
Trend of neonatal deaths in Uttar Pradesh, 2011–2020

Cumulatively, from 2011 to 2020, implementation of ReMiND intervention in UP would result in a reduction of 312 maternal and 149,468 neonatal deaths during the 10-year period (Table 3). This implies a reduction of 0.2% maternal and 5.3% neonatal deaths (Figs. 3, 4). The reduction in maternal illnesses during pregnancy and neonatal illnesses were 9.11 and 1.9% respectively, between the intervention and counterfactual scenarios. This resulted in increase in 2,231,275 life years and reduction of 4,127,529 DALYs.

### Cost effectiveness

**Fig. 5:**
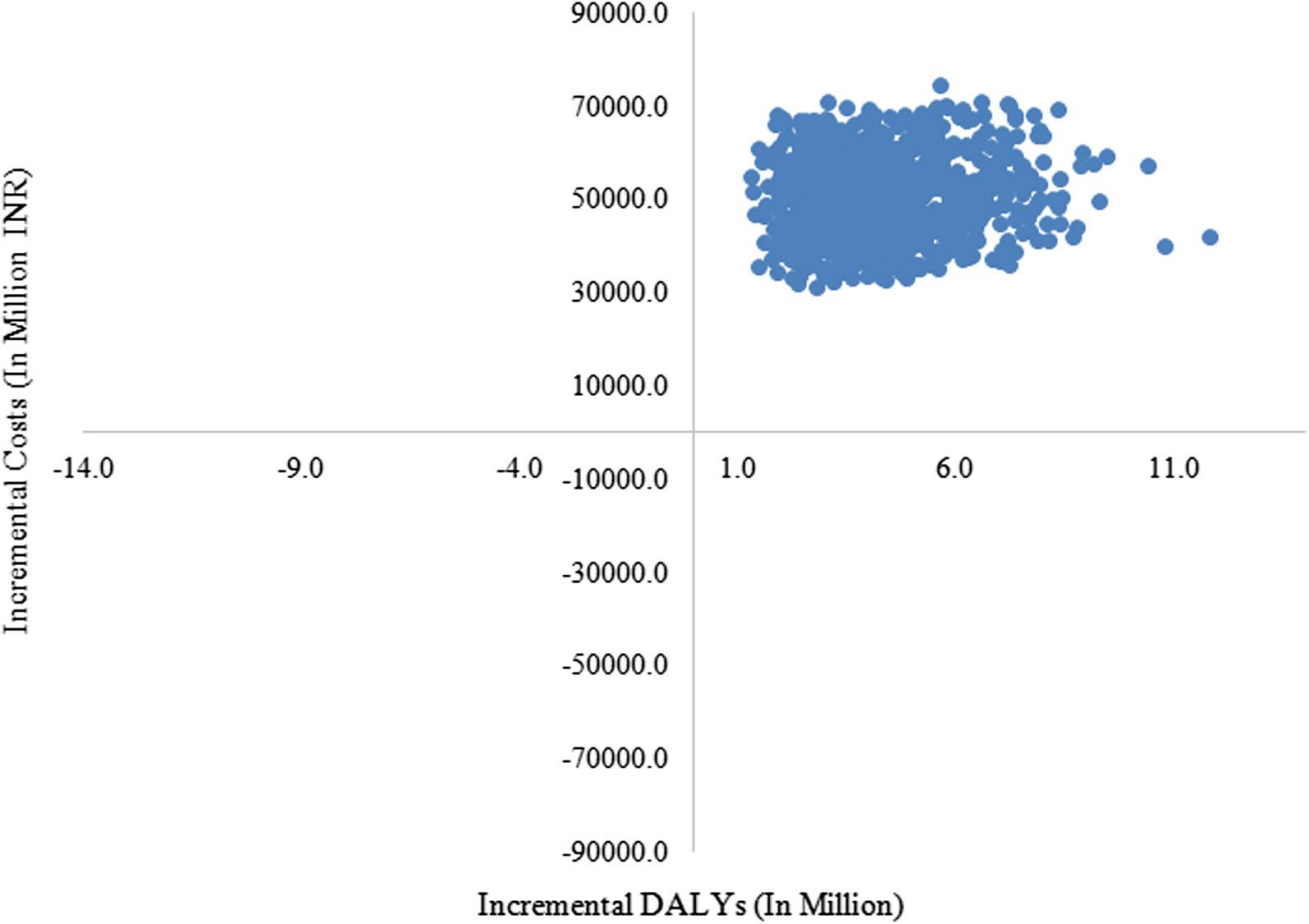
Cost effectiveness plane with incremental cost effectiveness ratios, health system perspective

**Fig. 6:**
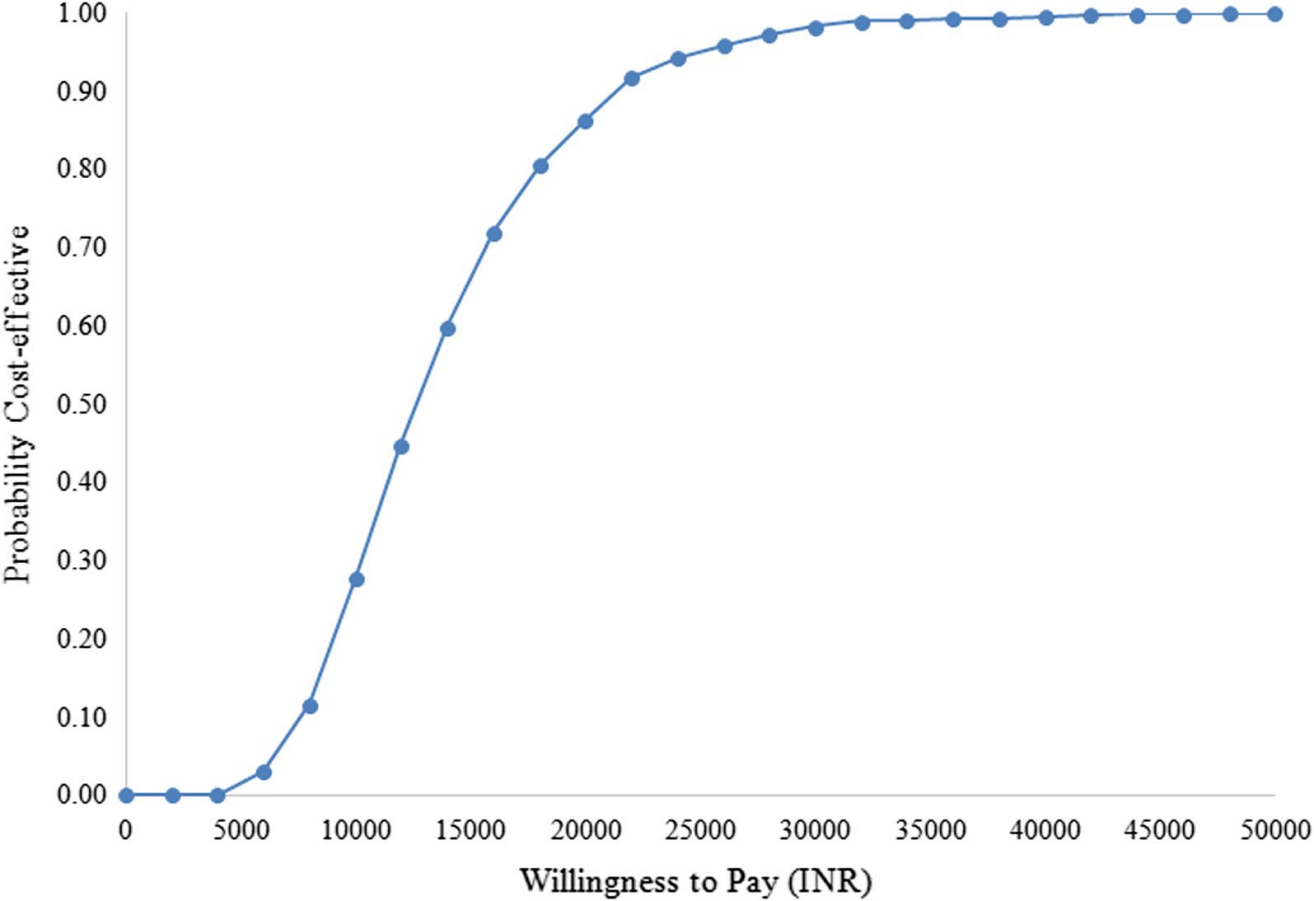
Cost effectiveness acceptability curve, health system perspective

We found the ReMiND intervention to be cost saving from the societal perspective (Table 3, Additional file 4: Figure S2 and Additional file 5: Figure S3). ReMiND intervention resulted in a cost saving of USD 90 per DALY averted USD 2569 per death averted (Table 3). From health system perspective, t incurs an incremental cost of INR 12,993 (USD 205) per DALY averted and INR 371,577 (USD 5866) per death averted (Table 3). Figure 5 shows results from simulations done as a part of probabilistic sensitivity analysis. The points in scatter plot indicates that considering all the uncertainties in the analysis, majority concentration of simulated results are in quadrant I of cost-effectiveness plane. This implies that ReMiND intervention has additional health benefits at an additional cost (Fig. 5). With a GDP per capita of nearly USD 1500 per capita, the ReMiND intervention for reducing maternal and neonatal mortality is very cost effective from Indian health system perspective. Accounting for all the uncertainties in the analysis, there is a 90% probability of ReMiND intervention to be cost-effective at willingness to pay threshold of USD 354 i.e. INR 22,500 which is only 23.6% of per capita GDP of India in 2016 (Fig. 6).

## Discussion

We undertook the present economic evaluation to compare the costs and consequences of implementing an mHealth intervention (ReMiND) in the existing set-up of routine health services, compared to routine maternal and child health services. In our analysis, we report findings from both health system and societal perspectives. We used per capita gross domestic product (GDP) of India as threshold for determining the cost-effectiveness. India had a GDP per capita of INR 88,440 (USD 1451.5) in 2013 [23, 69]. Our analysis shows that ReMiND implementation costs the Government of Uttar Pradesh an additional USD 205 (INR 12,993) per DALY averted. There is 90% probability of ReMiND intervention to be cost-effective at willingness to pay threshold of USD 354 i.e. INR 22,500 which is only 23.6% of per capita GDP of India in 2016. The Disease Control Priority 3 advises against the singular use of per capita GDP as the cost effectiveness threshold and rather recommends the comprehensive application of principles of equity and extended cost-effectiveness analysis while deciding about adoption of an intervention. It also highlights that the most cost effective interventions are the primary or preventive care interventions that prevent people from falling ill and seeking tertiary care. ReMiND program is one such intervention with the focus on enhancing the coverage of preventive care during pregnancy and early identification and referral for the complications [22]. ReMiND program become cost saving from a societal perspective. From a societal perspective, there is 88% probability of ReMiND intervention to be cost-effective if there is no difference in the costs of two scenarios (refer Additional file 4: Figure S2 and Additional file 5: Figure S3). We found that the improvements in counselling as a result of mHealth through ASHA workers, lead to generation of demand for preventive as well as curative care. Both preventive and curative care utilization ultimately leads to an increase in health system cost of health care. However, our modelled findings show that the increase in preventive services lead to a reduction of illness during pregnancy—such as anemia and hypertension, after child-birth and during neonatal period. Overall, from a health system perspective, an incremental cost of USD 982 million was incurred in the intervention setting. However, from societal perspective, there was an overall cost saving of USD 425 million. Despite a difference in increase (25.7%) in care being sought for such illnesses in intervention area [56], a reduction in number of illness episodes and OOPE resulted in these cost savings. A reduction in the occurrence of illnesses was due to increased uptake of preventive services—such as IFA supplementation, health monitoring through ANC care, whereas reduction in OOPE was because of increased utilization of public sector facilities both for preventive and curative care. Together these two reasons offset the increase in costs of increased utilization. As a result, the overall costs for preventive and curative care were higher in the counterfactual scenario of routine MNCH services rather than the mHealth intervention scenario (Table 3). In terms of health gains, ReMiND averted 3.1 million maternal illnesses and 37,337 neonatal illnesses. This translates to reduction of 312 maternal deaths, 0.15 million neonatal deaths and 4.1 million DALYs. In intervention setting, our model estimated more number of neonatal deaths averted corresponding to neonatal illnesses averted. This is due to the fact that we assumed in our model, prematurity which is one of the major cause of neonatal death either leads to death or major/minor disability but does not leads to illness episode.

A number of estimates for cost effectiveness of individual child health interventions are available such as haemophilus influenza type ‘b’ vaccine [70], insecticide treated bednets for malaria [71, 72], HIV preventive interventions for maternal to child transmission etc. [73]. In terms of findings from developing country context, ReMiND is as cost effective as some of the well-known child health interventions such as vitamin A and zinc fortification, measles immunization, case management of pneumonia and oral rehydration therapy (USD 24) [74]. Within the literature from India, ReMiND (USD 205) is less cost effective than measles (USD 13.8), hepatitis B (USD 31), HPV vaccination (USD 1.1) [26, 75] and rotavirus vaccination (USD 139) [76]. However, it is more cost effective than some other vaccines against cholera (USD 595–1310), typhoid (USD 227–621) and haemophilus influenza type ‘b’ (USD 363) [70].

Our study has several methodological strengths. Firstly, our decision model is plausible in terms of program implementation design, care-seeking and health care delivery system. A decision tree was considered appropriate than any other modelling method, such as Markov model, considering the acute nature of most maternal and childhood illnesses. The decision tree models have been used in cost effectiveness studies across the globe specifically to calculate the incremental cost effectiveness ratios of various maternal and newborn programs. For instance, to calculate cost effectiveness of ‘Integrated Management of Newborn and Childhood Illnesses’ program in India [37]; group B streptococcal vaccine immunization to prevent neonatal sepsis and meningitis in Sub Saharan Africa [77]; voucher scheme combined with obstetrical quality improvements as a part of quasi experimental results from Uganda [78] and maternal and Child health voucher scheme in Myanmar [79].

Secondly, almost all the values for parameters were sourced from local Indian context. Most of these values have been drawn from findings of a quasi-experimental study which enhances the internal validity of our estimations [20]. Thirdly, a comprehensive costing analysis was undertaken to analyse all costs involved with intervention and counterfactual scenarios. Some costs were obtained from the published literature from the neighbouring states where not much difference in infrastructure is expected [43, 45].

### Limitations

The present analysis relied on modelled estimation of reduction in mortality and morbidity as a result of improvements in uptake of preventive services, based on robust evidence on such downstream benefits. It is very difficult to measure changes in maternal mortality and as a result we had to resort to modelling to document the same. Several analysis based on the LiST tool have shown that the modelled long-term benefits in terms of reduction in mortality are quite similar to those where empirical observations are available [80]. There were several reasons for preferring our own decision model over LiST. First, LiST model uses set of evidence based on systematic reviews about effect of m-health interventions on coverage of various preventive and curative services. While the validity of LiST model assumptions is not to be questioned, the context of a given intervention remains ‘central’ for assessment of its effectiveness. In this regard, while the LiST model uses global evidence on impact of interventions, the current intervention was implemented in Uttar Pradesh state of India—a setting which has much poorer maternal and child health indicators. The potential of any intervention to create an impact is dependent on the baseline situation. Hence, it might be better to rely on estimates from local settings if available and use it to populate the model. Secondly, the data on assumptions to link proximal effectiveness estimates (such as coverage of health services) to long term outcomes (including morbidity and mortality) were derived using studies from India. We also varied these assumptions and undertook a probabilistic sensitivity analysis to check the robustness of our results to variations in parameter uncertainty. Thirdly, LiST provides framework for modelling long term outcomes by populating a model on a set package of interventions. However, this leaves relatively little user flexibility to incorporate the specific aspects of a given intervention which may be different to the package of interventions which were considered while populating the LiST model. We used a previously validated model to assess the cost-effectiveness of Integrated Management of Neonatal and Child Illnesses in public health setting in India [37], and adapted it to incorporate the specifics of ReMiND program so that it becomes much more generalized to Indian settings.

Modelling the effect of multiple interventions which have effect on the same outcome is an area which needs methodological development. It is not clear whether the effect of several interventions will be additive, multiplicative or otherwise. However, in our case we have primarily relied on modelling the impact of IFA supplementation on anaemia during pregnancy; and ANC visits on hypertension during pregnancy. Each of these morbidities is then modelled separately for complications and mortality as a result. Secondly, the effect of improved care-seeking is directly on the case-fatality rate, rather than reduction of any illness. Hence we believe that this limitation could not have confounded our analysis. We do acknowledge that such modelled outcomes will result only if the supply-side of health system is able to meet the demand generated through the interventions like ReMiND. Hence, while the findings of the study recommend up scaling of ReMiND on grounds of efficiency, it is equally important to continue to focus of health system strengthening so that the supply of services match up to the demand generated, both in terms of quantity and quality. Secondly, the cost of scaling up will also need to be assessed. The cost of scaling up ReMiND intervention from two blocks in Kaushambi to 821 blocks in state was estimated using two case scenarios. This included a scenario in which the existing human resource in state health department was used for monitoring and supervisory activities, and second, if an additional supervisor was recruited in every block as was the case in original ReMiND pilot implementation in Kaushambi district. We found that using existing human resources, ReMiND scale up in UP state would cost INR 876 million (US$ 13.8 million) annually, implying a cost per pregnant woman of INR 175.3 (US $2.77). Similarly, in case of additional supervisory cadre was created, the overall annual cost and cost per pregnant woman would be INR 993 million (US $15.7 million) and INR 198.8 (US $ 3.14) respectively [40]. However, these costs were estimated in ideal conditions without considering any bottlenecks in the implementation of programme which may deviate to some extent in the real life situations. We also acknowledge that the scale up of such community health intervention depends on a number of unforeseen social and political factors which are beyond the control of the researchers. We have estimated costs in the ideal conditions without considering any bottlenecks in the implementation of programme.

As per the guidelines of Financial Management Group of National Health Mission—India’s flagship health program, it is recommended to increase the budget in program implementation plan of high priority districts by 10–15% annually [81]. Since the cost for scale up of ReMiND intervention is 6% of the total budget allotted to ‘Maternal and child health’ line item under the NRHM budget of UP state, the intervention appears financially sustainable. However, scale up of such community health interventions is social and political factors which are beyond the scope of present research. Changes in political and administrative structure also affects introduction of newer programs. Besides, scale-up of ReMiND intervention would also involve several rounds of training the health workers in use of technology, as well as their supportive supervision. We acknowledge that these uncertainties could not be accounted for in our analysis. For assessing the impact of the intervention, we have used different datasets of 2 time-periods to represent baseline and end-line period. The endline survey was designed to be similar to the baseline in order to avoid any bias. However, since the intervention was not randomly assigned, there could be possibility of confounding due to individual or community level characteristics. We used two approaches, i.e. matching of control with intervention blocks, and matching of individuals within these two areas in order to avoid confounding which are explained in detail in our paper which reports on impact of ReMiND intervention [20] and is also explained in the Additional file 6: Appendix S3. Hence, we believe that the impact assessment is robust.

Finally, in terms of costing, we relied on collection of cost data retrospectively. This implies that we collected data of resources spent for services delivered around 4 years old. This could have lead to a recall bias in usual scenario. However, since the entire data on resource use and expenditure was digitized, there is little chance of any recall bias. Moreover, since the intervention is still continuing, time-motion observations were made in order to develop statistics for apportioning joint costs, which are explained in the cost-analysis paper [40]. However, we do acknowledge that we could not directly interview a few officials who were involved in designing of the software, and whose inputs could have improved the estimates further. We also were not able to include wage or productivity loss as indirect expenditures while calculation of societal costs. In present study, the value for money for mHealth interventions in high burden settings like Kaushambi was calculated. However, there is a need to take further analysis to assess cost effectiveness in low burden settings. Since the state level estimates were used in model instead of two intervention blocks, the results are more generalizable to entire state.

## Conclusions

Overall, the findings of our study suggest strongly that the mHealth intervention as part of the ReMiND intervention is very cost effective from Indian health system’s viewpoint, and cost saving from a societal perspective, and should be considered for replication elsewhere in India. Such interventions for generating demand through community health worker programs would need to be matched with similar strengthening of the health system which is able to meet the increase in demand for services, both in terms of quantity and quality.

## Additional files

**Additional file 1: Figure S1.** A: Decision Model for Cost-effectiveness (Child health) study of m-health application for ASHA workers as part of ReMiND program. B: Outcome Model for Cost-effectiveness study of m-health application for ASHA workers as part of the ReMiND program. C. Decision Model for Cost-effectiveness (Maternal health) study of m-health application for ASHA workers as part of ReMind project.

**Additional file 2: Appendix S1.** CHEERS checklist.

**Additional file 3: Appendix S2.** Cost Analysis of ReMiND program.

**Additional file 4: Figure S2.** Cost Effectiveness Plane with Incremental Cost Effectiveness Ratios, Societal Perspective.

**Additional file 5: Figure S3.** Cost Effectiveness Acceptability Curve, Societal Perspective.

**Additional file 6: Appendix S3.** Impact assessment of ReMiND program.
