# Supplementary material for: Cost effectiveness of mHealth intervention by community health workers for reducing maternal and newborn mortality in rural Uttar Pradesh, India
Source: Cost Eff Resour Alloc. 2018 Jun 25;16:25. doi: 10.1186/s12962-018-0110-2 (PMC6020234; doi:10.1186/s12962-018-0110-2)

Additional file 1: Figure S1**:**

**Figure S1A: Decision Model for Cost-effectiveness (Child health) study of m-health application for ASHA workers as part of ReMiND program**

**
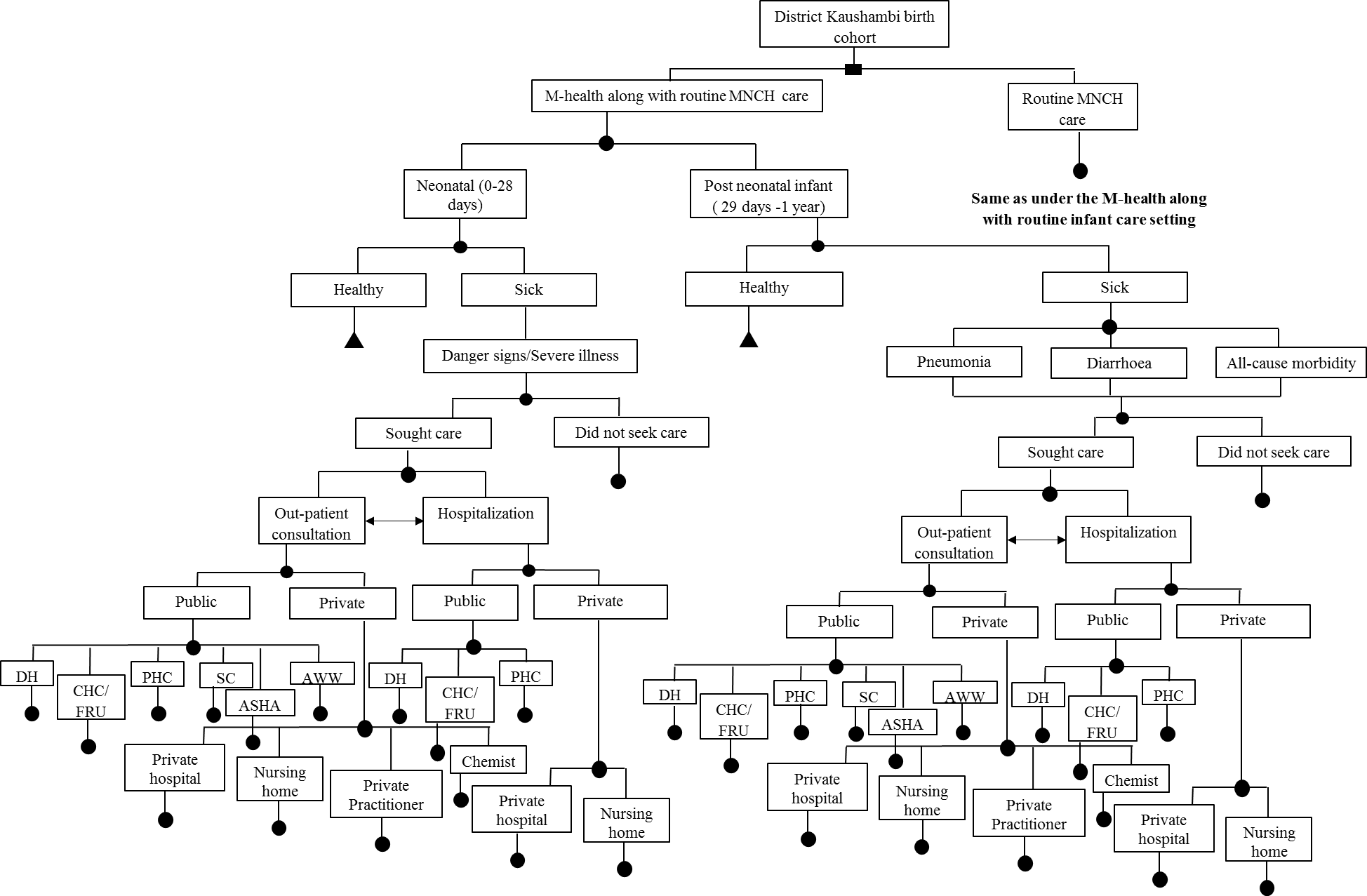
**

**Note**: DH= District Hospital, CHC= Community health centre, FRU= First referral unit, PHC= Primary health centre, SC= sub-centre, ASHA= Accredited social health activist, AWW= Anganwadi worker, MNCH= Maternal neonatal and child health. Cycle repeated for 15 birth cohorts.

**Figure S1B: Outcome Model for Cost-effectiveness study of m-health application for ASHA workers as part of the ReMiND program**


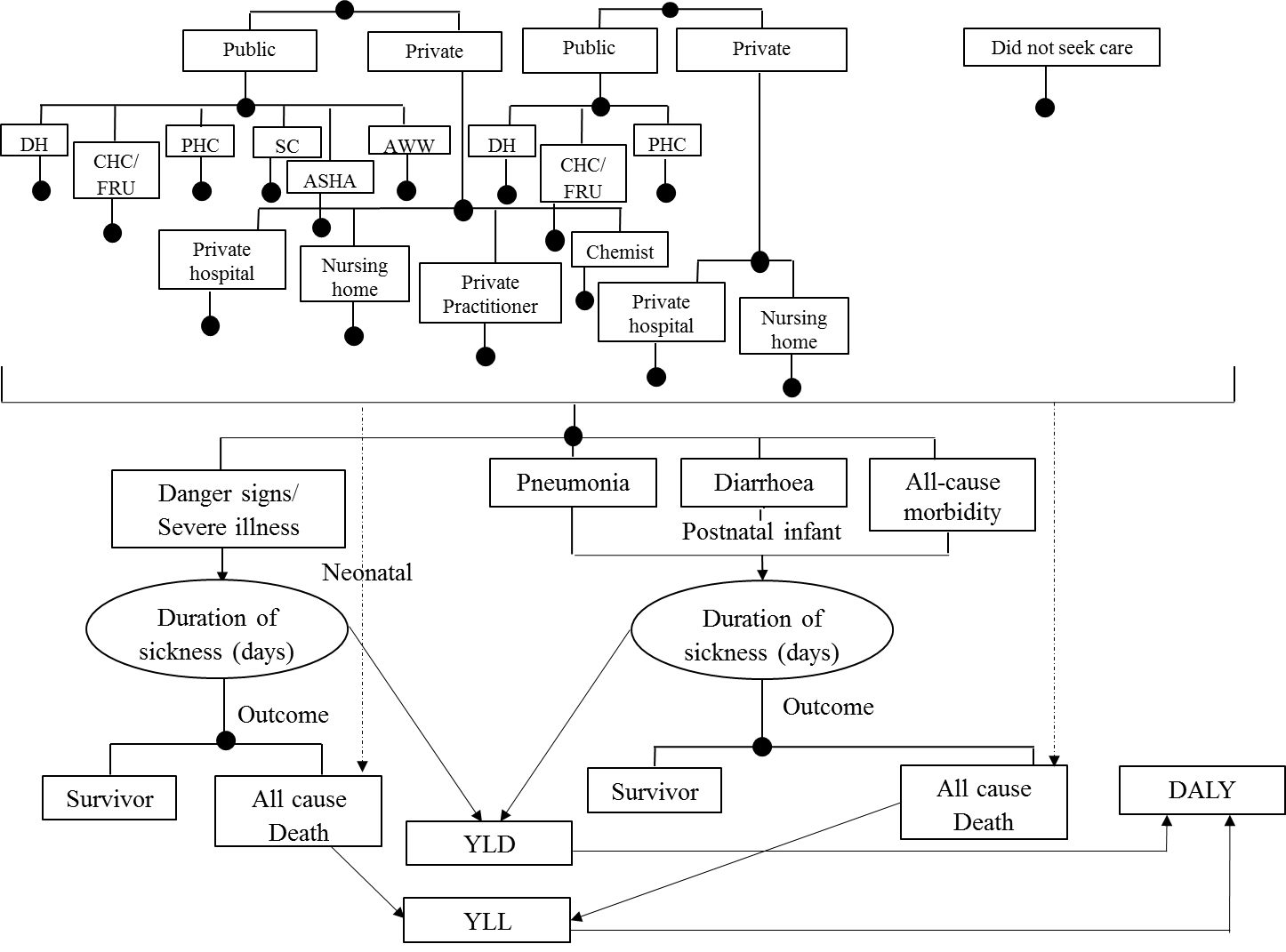


**Note**: In continuation to the model 1, outcome model describes the probable scenarios after a neonatal or post-neonatal infant had been treated or not (irrespective of type of health facility). YLD= Years of life lived with disability, YLL= Years of life lost due to premature mortality, DALY= Disability adjusted life years.

(Source: figure already published and assessable at reference, Prinja S, Nimesh R, Gupta A, Bahuguna P, Thakur JS, Gupta M, et al. Impact assessment and cost-effectiveness of m-health application used by community health workers for maternal, newborn and child health care services in rural Uttar Pradesh, India: a study protocol. Global health action. 2016;9)

**Figure S1C: Decision Model for Cost-effectiveness (Maternal health) study of m-health application for ASHA workers as part of ReMind project**


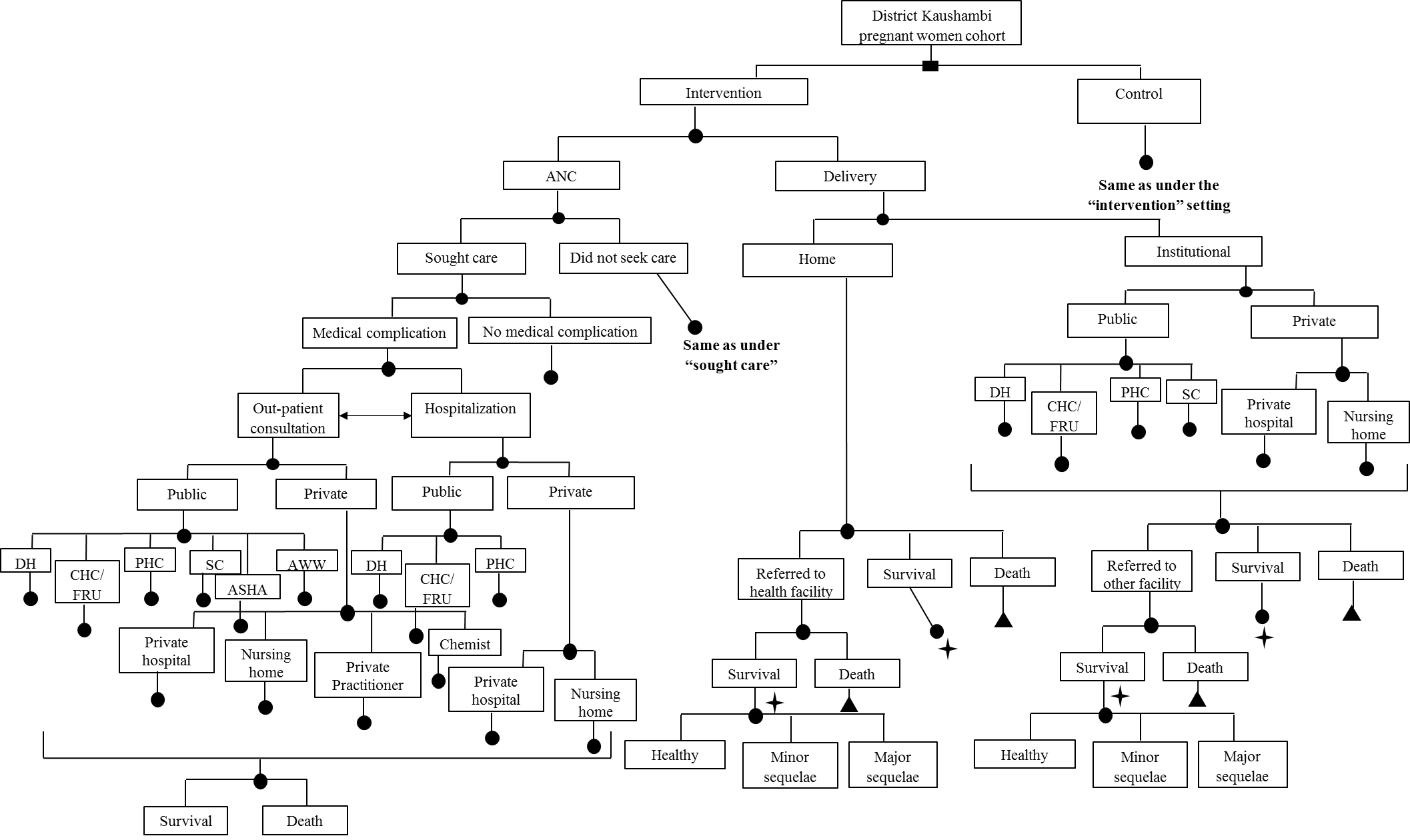

Supplement: Supplementary file 1 — Additional file 1: Figure S1. A: Decision Model for Cost-effectiveness (Child health) study of m-health application for ASHA workers as part of ReMiND program. B: Outcome Model for Cost-effectiveness study of m-health application for ASHA workers as part of the ReMiND program. C. Decision Model for Cost-effectiveness (Maternal health) study of m-health application for ASHA workers as part of ReMind project. [file 12962_2018_110_MOESM1_ESM.docx]
