# Supplementary material for: Cost effectiveness of mHealth intervention by community health workers for reducing maternal and newborn mortality in rural Uttar Pradesh, India
Source: Cost Eff Resour Alloc. 2018 Jun 25;16:25. doi: 10.1186/s12962-018-0110-2 (PMC6020234; doi:10.1186/s12962-018-0110-2)
Supplement: Supplementary file 2 — Additional file 2: Appendix S1. CHEERS checklist. [file 12962_2018_110_MOESM2_ESM.docx]

**Additional file 2: Appendix S1: CHEERS checklist—Items to include when reporting economic evaluations of health interventions**

| **Section/item** | | | **Item No** | | **Recommendation** | **page No/ line No** |
| --- | --- | --- | --- | --- | --- | --- |
| **Title and abstract** | | |  | |  |  |
| Title | | | 1 | | Identify the study as an economic evaluation or use more specific terms such as “cost-effectiveness analysis”, and describe the interventions compared. | 1/1 |
| Abstract | | | 2 | | Provide a structured summary of objectives, perspective, setting, methods (including study design and inputs), results (including base case and uncertainty analyses), and conclusions. | 2-3/25-52 |
|  | | | | | | |
| Background and objectives | | | 3 | | Provide an explicit statement of the broader context for the study. | 6/110-124 |
|  | | |  | | Present the study question and its relevance for health policy or practice decisions. | 6/110-124 |
| **Methods** | | | | | | |
| Target population and subgroups | 4 | | | Describe characteristics of the base case population and subgroups analyzed, including why they were chosen. | | 8/158-161 |
| Setting and location | 5 | | | State relevant aspects of the system(s) in which the decision(s) need(s) to be made. | | 7/145-149 |
| Study perspective | 6 | | | Describe the perspective of the study and relate this to the costs being evaluated. | | 10/209,  Supplementary Information S2 |
| Comparators | 7 | | | Describe the interventions or strategies being compared and state why they were chosen. | | 9/178-190 |
| Time horizon | 8 | | | State the time horizon(s) over which costs and consequences are being evaluated and say why appropriate. | | 10/194-208 |
| Discount rate | 9 | | | Report the choice of discount rate(s) used for costs and outcomes and say why appropriate. | | 11/220-231 |
| Choice of health outcomes | 10 | | | Describe what outcomes were used as the measure(s) of benefit in the evaluation and their relevance for the type of analysis performed. | | 10-11/213-219 |
| Measurement of effectiveness | 11a | | | *Single study-based estimates:* Describe fully the design features of the single effectiveness study and why the single study was a sufficient source of clinical effectiveness data. | | 15/311-319,  Supplementary Information S1,  Proximal coverage estimates were derived from the parameters taken from the impact assessment study. The reference is given from study (1) |
|  | 11b | | | *Synthesis-based estimates:* Describe fully the methods used for identification of included studies and synthesis of clinical effectiveness data. | | 16/342-351,  An extensive literature review along with expert opinions were carried out to find the value of effectiveness parameters used in our decision model (Mentioned in table 1) |
| Measurement and valuation of preference based outcomes | 12 | | | If applicable, describe the population and methods used to elicit preferences for outcomes. | | 11/237-238,  For preference based outcomes, disability rates were taken from the Global Burden of disease data. |
| Estimating resources and costs | 13a | | | *Single study-based economic evaluation:* Describe approaches used to estimate resource use associated with the alternative interventions. Describe primary or secondary research methods for valuing each resource item in terms of its unit cost. Describe any adjustments made to approximate to opportunity costs. | | 12-13/240-271,  Supplementary Information S2.  Economic costing using a bottom up approach was used, details are given in the report cited as reference (2), |
|  | 13b | | | *Model-based economic evaluation:* Describe approaches and data sources used to estimate resource use associated with model health states. Describe primary or secondary research methods for valuing each resource item in terms of its unit cost. Describe any adjustments made to approximate to opportunity costs. | | 13-14/274-291  Table 1- shows data sources and Supplementary appendix S2. |
| Currency, price date, and conversion | 14 | | | Report the dates of the estimated resource quantities and unit costs. Describe methods for adjusting estimated unit costs to the year of reported costs if necessary. Describe methods for converting costs into a common currency base and the exchange rate. | | 12/43-247 |
| Choice of model | 15 | | | Describe and give reasons for the specific type of decision-analytical model used. Providing a figure to show model structure is strongly recommended. | | Decision tree has been added as Supplementary Figure S1  26/ 548-561 |
| Assumptions | 16 | | | Describe all structural or other assumptions underpinning the decision-analytical model. | | Table 1 |
| Analytical methods | 17 | | | Describe all analytical methods supporting the evaluation. This could include methods for dealing with skewed, missing, or censored data; extrapolation methods; methods for pooling data; approaches to validate or make adjustments (such as half cycle corrections) to a model; and methods for handling population heterogeneity and uncertainty. | | Supplementary Appendix S1 and S2.  Population heterogeneity was taken care of by using data from UP state rather than intervention blocks for modeling estimates like data on coverage, morbidity and mortality. Also, in the effectiveness study, control areas were matched with the intervention areas along with matching of beneficiaries in two areas. Thus, potential heterogeneity does not affect the estimates.  Also, the cost data was inflated and deflated as required in the calculations. |
| **Results** | | | | | | |
| Study parameters | | 18 | | Report the values, ranges, references, and, if used, probability distributions for all parameters. Report reasons or sources for distributions used to represent uncertainty where appropriate. Providing a table to show the input values is strongly recommended. | | Table 1 |
| Incremental costs and outcomes | | 19 | | For each intervention, report mean values for the main categories of estimated costs and outcomes of interest, as well as mean differences between the comparator groups. If applicable, report incremental cost-effectiveness ratios. | | Table 3 |
| Characterizing uncertainty | | 20a | | *Single study-based economic evaluation:* Describe the effects of sampling uncertainty for the estimated incremental cost and incremental effectiveness parameters, together with the impact of methodological assumptions (such as discount rate, study perspective). | | 21/453-457,  The estimates of effectiveness parameters were derived from the analysis of primary data from single study. It also gave standard error estimates based on sampling variations. This was tested in Probabilistic Sensitivity Analysis (PSA).  In the model based economic evaluation, the values from various other Indian studies were taken. Results should be viewed in light of these differences |
|  |  | 20b | | *Model-based economic evaluation:* Describe the effects on the results of uncertainty for all input parameters, and uncertainty related to the structure of the model and assumptions. | |  |
| Characterizing heterogeneity | | 21 | | If applicable, report differences in costs, outcomes, or cost-effectiveness that can be explained by variations between subgroups of patients with different baseline characteristics or other observed variability in effects that are not reducible by more information. | | The subgroup analysis was not done in the study. |
| **Discussion** | | | | | | |
| Study findings, limitations, generalizability, and current knowledge | | 22 | | Summarise key study findings and describe how they support the conclusions reached. Discuss limitations and the generalisability of the findings and how the findings fit with current knowledge. | | In present study, the value for money for mHealth interventions in high burden settings like Kaushambi was calculated. However, there is a need to take further analysis to assess Cost effectiveness assessment in low burden settings. Since the state level estimates were used in model instead of two intervention blocks, the results are more generalisable to entire state  Summary=24/518-522  Limitation=27-30/568-656 |
| **Other** | | | | | | |
| Source of funding | | 23 | | Describe how the study was funded and the role of the funder in the identification, design, conduct, and reporting of the analysis. Describe other non-monetary sources of support. | | 33/701-704 |
| Conflicts of interest | | 24 | | Describe any potential for conflict of interest of study contributors in accordance with journal policy. In the absence of a journal policy, we recommend authors comply with International Committee of Medical Journal Editors recommendations. | | 32/692 |

References

1. Prinja S, Nimesh R, Gupta A, Pankaj B, Gupta M, Thakur JS. Impact of m-Health application used by community health volunteers for improving utilization of maternal, newborn and child health care (MNCH) services in a rural area of Uttar Pradesh, India. Trop Med Int Health. 2017. doi:10.1111/tmi.12895.

2. Prinja S, Gupta A, Bahuguna P, Nimesh R. Cost analysis of implementing m-health intervention for maternal, newborn & child health care through community health workers: Assessment of ReMiND Program in Uttar Pradesh, India. [Internet]. Chandigarh: School of Public Health, Post Graduate Institute of Medical Education and Research; 2015 [cited 2017 July 10]. Available from: <http://www.healtheconomics.pgisph.in/admin/publication/cost_analysis_of_ReMiND_project.pdf>.
