# Supplementary material for: Cost effectiveness of mHealth intervention by community health workers for reducing maternal and newborn mortality in rural Uttar Pradesh, India
Source: Cost Eff Resour Alloc. 2018 Jun 25;16:25. doi: 10.1186/s12962-018-0110-2 (PMC6020234; doi:10.1186/s12962-018-0110-2)
Supplement: Supplementary file 3 — Additional file 3: Appendix S2. Cost Analysis of ReMiND program. [file 12962_2018_110_MOESM3_ESM.docx]

**Additional file 3: Appendix S2: Cost Analysis of ReMiND program**

To assess the annual & unit cost of ReMiND program and its scale up in Uttar Pradesh state in India, economic costing was done from the health system and societal perspectives. Health system costs’ are described as the costs of resources which are consumed to provide particular service which in our case was routine maternal and newborn health care. The costs on resources like building/space, salaries of human workforce, equipments, medicines, consumables, overheads etc are included. Whereas, societal costs captures all the costs incurred by health system and society i.e. households comprehensively. Household costs are comprised of out-of-pocket expenditures (OOPE) incurred for purchasing medicines, any medical or surgical procedures, boarding, lodging, transportation and the indirect costs in terms of productivity loss to the household/economy as a result of absenteeism due to treatment of illness.

We included two cost heads i.e. start up cost and implementation cost. All resources used during these phases were quantified and valued. The identified cost centres are shown in the figure provided below. Start up costs included all the capital costs incurred during the initial preplanning and planning phase. This was categorised as costs levied on modules’ development and piloting, development and maintenance of software, equipment cost, mobile phones and overhead costs. Apart from these costs; pre-planning meetings, trainings of ASHAs & their supervisors and translation of modules into local dialect were also taken as capital costs because the effect of these inputs were likely to last for the life of the program. All the capital costs were apportioned in terms of their time value devoted in ReMiND program out of all the programs running simultaneously by the implementing agencies. The implementation costs included recurrent costs like salaries of human resources, travel, internet usage charges for mobile application, utilities like office rent, electricity, telephone bills & internet bills and stationary & printing. Apart from costs spent by implementing agencies, additional costs incurred by district health system were also estimated. It included three components – first, health system cost on routine program implementation; second, incremental cost of service delivery due to increased utilization of MNCH services as the result of intervention and third, incremental effect of intervention on ASHAs payment in terms of incentives. For program implementation, the extra cost of monthly meetings at the block levels, quarterly meetings at district level and bi-annual meetings at the state level were calculated by apportioning the time devoted by government officials in meetings for review of ASHAs’ performance under the program. The incremental time spent on monitoring the mHealth intervention was calculated by interviewing all the officials from the state health system who were directly or indirectly involved in the monitoring and supervision of the program in the intervention and control areas. The monitoring and supervisory activities were carried out at block, district and state level in form of review meetings at monthly, quarterly and biannual intervals. The annual time spent by various officials specifically on review of ReMiND program was calculated and the salaries were proportioned accordingly to see what incremental cost goes to the monitoring activity of the program.

The costs in dollars were converted to Indian National Rupee (INR) by applying the dollar conversion rates given by the US Internal Revenue Service for a particular year of purchase of equipment. The converted rates were then inflated from the year of purchase to the current value of product in 2015 by applying Consumer Price Index in India. These inflated values of capital were then annualized as per the average life of utilization of the product at a discount rate of 3%. The annual maintenance rates for capital items as given by the implementing agency were used in calculations.

 For calculation of scale up costs, two case scenarios were assumed. First, if the available human resource in the health system could be utilised for monitoring and supervision of this intervention. Second, a separate supervisory cadre like sector facilitators in ReMiND program were employed at block level. In Uttar Pradesh, Block Community Managers and Health Education Officers are employed at block and district levels respectively to coordinate and supervise ASHAs’ performance by the government.

The annual cost for rolling out ReMiND in two blocks of district Kaushambi was INR 12.1 million (US $191,894). Out of this, start up cost and implementation cost contributed 9% and 91% of the total annual cost respectively. Government health system contributed 4.8% of the total implementation cost. The unit costs of implementing ReMiND program were INR 31.4 (US $ 0.49) per capita and INR 1294 (US $ 20.5) per registered pregnant women.

The scale up would cost INR 876 million (US$ 13.8 million) to state with INR 175.3 (US $2.77) per pregnant women if existing human resources for monitoring and supervision is used. However, government has to spend around INR 993 million (US $15.7 million) with INR 198.8 (US $ 3.14) per pregnant woman if additional human resource was recruited for monitoring of ASHAs in every block of state. The detailed description of the costing report is available elsewhere as the report (1).

Reference

1. Prinja S, Gupta A, Bahuguna P, Nimesh R. Cost analysis of implementing m-health intervention for maternal, newborn & child health care through community health workers: Assessment of ReMiND Program in Uttar Pradesh, India. [Internet]. Chandigarh: School of Public Health, Post Graduate Institute of Medical Education and Research; 2015 [cited 2017 July 10]. Available from: <http://www.healtheconomics.pgisph.in/admin/publication/cost_analysis_of_ReMiND_project.pdf>.
