# Supplementary material for: Cost effectiveness of mHealth intervention by community health workers for reducing maternal and newborn mortality in rural Uttar Pradesh, India
Source: Cost Eff Resour Alloc. 2018 Jun 25;16:25. doi: 10.1186/s12962-018-0110-2 (PMC6020234; doi:10.1186/s12962-018-0110-2)
Supplement: Supplementary file 4 — Additional file 4: Figure S2. Cost Effectiveness Plane with Incremental Cost Effectiveness Ratios, Societal Perspective. [file 12962_2018_110_MOESM4_ESM.docx]

**Additional file 4: Figure S2: Cost Effectiveness Plane with Incremental Cost Effectiveness Ratios, Societal Perspective**
