# Supplementary material for: Cost effectiveness of mHealth intervention by community health workers for reducing maternal and newborn mortality in rural Uttar Pradesh, India
Source: Cost Eff Resour Alloc. 2018 Jun 25;16:25. doi: 10.1186/s12962-018-0110-2 (PMC6020234; doi:10.1186/s12962-018-0110-2)
Supplement: Supplementary file 6 — Additional file 6: Appendix S3. Impact assessment of ReMiND program. [file 12962_2018_110_MOESM6_ESM.docx]

**Additional file 6: Appendix S3: Impact assessment of ReMiND program**

To assess the impact of mHealth intervention (also called ReMiND program) in reducing maternal and newborn deaths in district Kaushambi of Uttar Pradesh state in India, we undertook a pre and post quasi experimental study. Two blocks other than two intervention blocks were selected as controls after matching for coverage of two indicators at baseline - ante natal care and institutional deliveries from the same district. The pre-intervention data was obtained from the Annual Health Survey (AHS) 2011 conducted by Ministry of Health and Family Welfare while for post-intervention coverage data, a household survey was carried out in four blocks of Kaushambi district in 2015. Mothers in two categories, first, those with child aged between 0 to 28 days (n=1053) and 29 days to 6 months (n=1391) were interviewed in each of the intervention and control areas. Mothers with child in age of 29 days to 6 months were interviewed for collecting information on socio-demographic characteristics; utilization of antenatal care services, institutional delivery and postnatal care; and data on occurrence of any morbidity during neonatal period, its care seeking and out of pocket expenditure. Women with the children in the age group of 12-23 months were interviewed for socio-demographic characteristics and immunization status of their infants. Propensity score matched samples in both age groups from intervention and control areas in pre-intervention and post-intervention periods were analysed using difference-in-difference method to estimate the impact of ReMiND intervention. A statistically significant increase in coverage IFA supplementation (12.70%), identification and self-reporting of illnesses/ complication during pregnancy (13.20%) and after delivery (19.5%) was observed in the intervention area as compared to the control area. The coverage of >=3 ANC visits, >=2 tetanus toxoid, ­ Full ANC, and ambulance usage also increased in the intervention area by 9.7%, 4.5%, 1% and 2.5% respectively, however, the change was statistically insignificant. We found a statistically insignificant change in quality of ANC care such as blood pressure, urine test, weight measurement and blood test during ANC care between the intervention and control area. The statistically significant parameters are described in the table below. The impact assessment of ReMiND program is already published in journal of ‘Tropical Medicine and International Health’ is available online and is cited for your reference (1).

S1 Table: Summary of the various health indicators after matching

| Indicator | Intervention | | | Control | | | Difference in difference |
| --- | --- | --- | --- | --- | --- | --- | --- |
|  | AHS 2011 | CEAHH 2015 | Change | AHS 2011 | CEAHH 2015 | Change |  |
| >=100 IFA consumption | 2.00% | 1.00% | -1.00% | 14.10% | 0.40% | -13.70% | 12.7% |
| Self-reporting of illnesses during Pregnancy | 40.40% | 84.80% | 44.40% | 45.50% | 76.70% | 31.20% | 13.20% |
| Self-reporting of illnesses after Delivery | 34.30% | 50.60% | 16.30% | 38.40% | 35.20% | -3.20% | 19.5% |

The out of pocket expenditures were also collected from the intervention and control households in the household survey. The m-health intervention may have had affected out of pocket expenditures overtime horizon of 10 years due to following reasons. First, we found a statistically significant increase in the proportion of women recognising early signs of danger during pregnancy and after delivery in intervention area. While this may result in an increase in OOP for outpatient care, it can reduce more severe complications and need for inpatient care. Second, we found that more women in intervention group were visiting public health facilities than those in control groups for maternal, newborn and childhood illnesses. Since the out of pocket expenditures in the public sector hospitals were lesser than the private sector health facilities, it can have a reduction in overall OOP expenditures. Thus, a cumulative effect of all these factors is likely to decrease the out of pocket expenditure in the intervention area.

To ensure the robustness of the effect estimation, we used the recommended methods for sample selection and data analysis to enhance the casual attribution of intervention’s effectiveness. First, the community development blocks from the same district which had closest coverage of full antenatal care and institutional delivery as compared to intervention block were selected as control area. Second, the propensity score matched (PSM) households from the control area were chosen to control for household level demand side factors which could influence service utilization. Third, the PSM data of intervention and control households was analyzed using Difference in Difference (DID) method in a quasi experimental study design. Such design is considered as robust to control the effect of observed and unobserved confounders in absence of randomized controlled trial study. Fourth, while there were several supply side interventions which were being implemented in district Kaushambi as part of National Health Mission to improve coverage of MNCH services, these interventions were implemented in all community development blocks of district Kaushambi uniformly. Hence the DID design for data analysis controls for the effect of these supply side interventions as well as unknown confounders. Finally, we acknowledge that there still may be chance of some unknown confounders which are beyond the statistical control.

Reference

1. Prinja S, Nimesh R, Gupta A, Pankaj B, Gupta M, Thakur JS. Impact of m-Health application used by community health volunteers for improving utilization of maternal, newborn and child health care (MNCH) services in a rural area of Uttar Pradesh, India. Trop Med Int Health. 2017. doi:10.1111/tmi.12895.
